# Camel whey protein hydrolysates induced G2/M cellcycle arrest in human colorectal carcinoma

**DOI:** 10.1038/s41598-021-86391-z

**Published:** 2021-03-29

**Authors:** Chandraprabha Murali, Priti Mudgil, Chee-Yuen Gan, Hamadeh Tarazi, Raafat El-Awady, Youssef Abdalla, Amr Amin, Sajid Maqsood

**Affiliations:** 1 Biology Department, College of Science, United Arab Emirates University, P.O. Box 15551, Al Ain, UAE; 2 Food, Nutrition and Health Department, College of Food and Agriculture, United Arab Emirates University, P.O. Box 15551, Al Ain, UAE; 3 Analytical Biochemistry Research Centre (ABrC), University Innovation Incubator Building, sains@usm campus, Universiti Sains Malaysia, 11900 Bayan Lepas, Penang Malaysia; 4 College of Pharmacy, University of Sharjah, Sharjah, UAE; 5 Department of Kinesiology, Michigan State University, East Lansing, MI 48824 USA

**Keywords:** Biotechnology, Cancer, Drug discovery, Gastroenterology

## Abstract

Camel milk has been gaining immmense importance due to high nutritious value and medicinal properties. Peptides from milk proteins is gaining popularity in various therapeutics including human cancer. The study was aimed to investigate the anti-cancerous and anti-inflammatory properties of camel whey protein hydrolysates (CWPHs). CWPHs were generated at three temperatures (30 ℃, 37 ℃, and 45 ℃), two hydrolysis timepoints (120 and 360 min) and with three different enzyme concentrations (0.5, 1 and 2 %). CWPHs demonstrated an increase in anti-inflammatory effect between 732.50 (P-6.1) and 3779.16 (P-2.1) µg Dicolfenac Sodium Equivalent (DSE)/mg protein. CWPHs (P-4.3 & 5.2) inhibited growth of human colon carcinoma cells (HCT116) with an IC_50_ value of 231 and 221 μg/ml, respectively. P-4.3 induced G2/M cell cycle arrest and modulated the expression of Cdk1, p-Cdk1, Cyclin B1, p-histone H3, p21 and p53. Docking of two peptides (AHLEQVLLR and ALPNIDPPTVER) from CWPHs (P-4.3) identified Polo like kinase 1 as a potential target, which strongly supports our in vitro data and provides an encouraging insight into developing a novel peptide-based anticancer formulation. These results suggest that the active component, CWPHs (P-4.3), can be further studied and modeled to form a small molecule anti-cancerous therapy.

## Introduction

The rapid development in the field of proteomics has portrayed the evolution of proteins from being a mere nutritional component to a major contributor to the modulation of body’s physiological functions. Food derived proteins render health benefits via the release of bioactive peptides upon gastrointestinal transformation mediated by hydrolysis induced by digestive enzymes, microbial proteases and fermentation^1^. Food derived peptides with anticancer and anti-inflammatory activities have been reported in a variety of food protein sources including milk, egg, fish, rice, soybean, pea and many others^2^. Bioactive peptides have been reported to regulate factors in carcinogenesis; oxidative stress and inflammation^3^. Therefore, their application in formulation of potential drugs, nutraceutical supplements and other pharmaceutical products has been widely investigated.

Bioactive peptides are highly selective to cancer cells and low toxicity to normal cells. Given their low molecular weight (~ 10^2^–10^3^ Da), they allow easy intercellular transportation and membrane interaction causing eventual cell death^4^. Anticancer peptides from marine life forms, reptiles, mammals and amphibians are studied^5^.

Due to its immense health benefits, camel milk has been receiving increasing global attention as one of the superfoods^6^. In the traditional medicine, local healers have claimed a therapeutic potential of a mixture of camel milk and urine to treat different types of cancer^7^. Unlike its bovine counterpart, camel milk is known to possess higher content of lactoferrin. Camel milk derived lactoferrin has been shown in vitro as a potent anticancer agent against colon cancer and breast cancer^8^. No detailed studies have been conducted to explore the anti-cancer effects of camel milk derived peptides. Our previous study reported potential anti-cancer capacity of camel whey protein hydrolysates against liver cancer in vitro^9^. Determination of anti-inflammatory and/or antioxidant properties has been proposed as a good indicator for screening anti-cancer agents^10^. With the ongoing advancement in the field of proteomics and bioinformatics, screening for novel anticancer peptides could emerge as a promising strategy to treat and/or prevent different types of cancers in the future. In this study we investigated the anti-proliferative property of the selected pepsin derived camel whey proteins hydrolysates on human cancer cells under in vitro conditions. Effects of hydrolysis variables such as E:S ratio, temperature, and reaction time were taken into consideration. Further potent hydrolysates underwent peptide sequencing followed by target protein binding studies to unravel the molecular mechanism.

## Results and discussion

### Effect of pepsin on the degree of hydrolysis (% DH) under different conditions including the E:S ratio, time and temperature

Analysing the release of free amino nitrogen (F-AN) during the proteolysis provide an overall extent of protein hydrolysis in comparison to the intact protein sample. In order to achieve required level of hydrolysis, the enzyme:substrate ratio, time and temperature of hydrolysis are among the main variables that need to be taken into consideration while producing the protein hydrolysates. DH (%) values of CWPHs are presented in Table 1. Overall, it was observed that DH values mainly increased with incubation time while no major effect of the enzyme concentration on %DH was evident. Similarly, no direct correlation between temperature and DH values were observed. For hydrolysates produced at 2% enzyme concentration, maximum DH value was expressed by hydrolysates P-1.1 produced at 30 ℃ for 2 h of hydrolysis. While for hydrolysates produced at 1% enzyme concentration, the highest DH was observed at 37 ℃ and for 0.5% enzyme concentration, the highest DH was found at 45 °C. Overall, the hydrolysate P-5.3 and P-4.1 displayed a higher yield of hydrolysis with DH values of ~ 57% and 52%, respectively, which was achieved after 2 h and 6 h of hydrolysis time and 0.5 and 1.0% enzyme concentration, respectively. The increase in degree of hydrolysis with time of hydrolysis might be attributed to higher availability of cleavage sites upon denaturation of the native proteins leading to higher reactivity of enzymes and consequently the breakdown of more peptide bonds^11^. The results obtained are in agreement with earlier investigation where DH values of hydrolysates generated from camel whey proteins using gastrointestinal enzyme at 37 ℃ was similar to the one reported here^12^. Studies related to the impact of pepsin digestion under variable conditions on the extent of hydrolysis of camel whey proteins are rather limited. However, a DH value of 20.5% has been reported upon peptic digestion of whole camel milk proteins for 30 min^13^. Those results were relatively higher than that observed by Salami, et al.^14^, where, hydrolysis of α-lactalbumin, a major whey protein, revealed a maximum DH value of ~ 10% at 37 ℃ for 180 min. These variations could simply be a result of using different camel milk protein matrix, as the whey used in the current study consisted of all major whey proteins like α-lactalbumin, serum albumins, lactoferrins and other minor whey proteins. That may explain the higher DH obtained in the present study.

**Table 1:** Effect of hydrolysis parameters on the degree of hydrolysis (DH %) and anti-inflammatory properties of peptic hydrolysates of camel whey proteins.

CWPHs	Hydrolysis parameters			DH (%)	Anti-inflammatory activity (DSE µg/mg PE)
	Temp (℃)	E (%)	Time (h)		
Whey	37	0	6		435.83 ± 46.45^a^
P-1.1	30	2	2	43.22 ± 0.65 ^fg^	3628.33 ± 168.78^jk^
P-1.2	37	2	2	16.67 ± 4.35^ab^	1909.83 ± 102.60^bcd^
P-1.3	45	2	2	14.67 ± 2.24^a^	1712.50 ± 57.66^b^
P-2.1	30	2	6	34.98 ± 4.01^e^	732.50 ± 0.01^a^
P-2.2	37	2	6	25.88 ± 0.89^c^	2912.50 ± 13.22^i^
P-2.3	45	2	6	36.36 ± 2.69^ef^	1872.50 ± 10.00^bcd^
P-3.1	30	1	2	26.77 ± 0.67^c^	2894.83 ± 32.97^hi^
P-3.2	37	1	2	37.09 ± 0.29^ef^	2605.00 ± 52.5^gh^
P-3.3	45	1	2	26.06 ± 1.26^c^	2305.00 ± 46.70^ef^
P-4.1	30	1	6	52.38 ± 1.06^hi^	2537.50 ± 15.00 ^fg^
P-4.2	37	1	6	48.33 ± 3.43 ^h^	1780.00 ± 97.50^bc^
P-4.3	45	1	6	23.47 ± 0.85^bc^	2072.50 ± 90.00^cde^
P-5.1	30	0.5	2	27.80 ± 0.25 ^cd^	2657.50 ± 250.00^ghi^
P-5.2	37	0.5	2	23.85 ± 1.64^c^	1767.50 ± 65.00^b^
P-5.3	45	0.5	2	57.99 ± 4.25^i^	3415.00 ± 122.50^j^
P-6.1	30	0.5	6	34.60 ± 2.56^de^	3779.16 ± 42.52 ^k^
P-6.2	37	0.5	6	25.16 ± 1.33^c^	2154.16 ± 70.23^de^
P-6.3	45	0.5	6	45.11 ± 6.16^gh^	1848.33 ± 145.39^bc^

Values represent mean ± standard deviations of three (n = 3) measurements. For increase in DH (%), values with different alphabets throughout the column are significantly (p < 0.05) different and for anti-inflammatory activity, values in a column within enzyme  concentration (%) are significantly (p < 0.05) different.

*DSE*:  diclofenac sodium equivalent, *PE*: protein equivalent.

### Peptide profiles of intact camel whey proteins and their peptic hydrolysates

**Figure 1:**
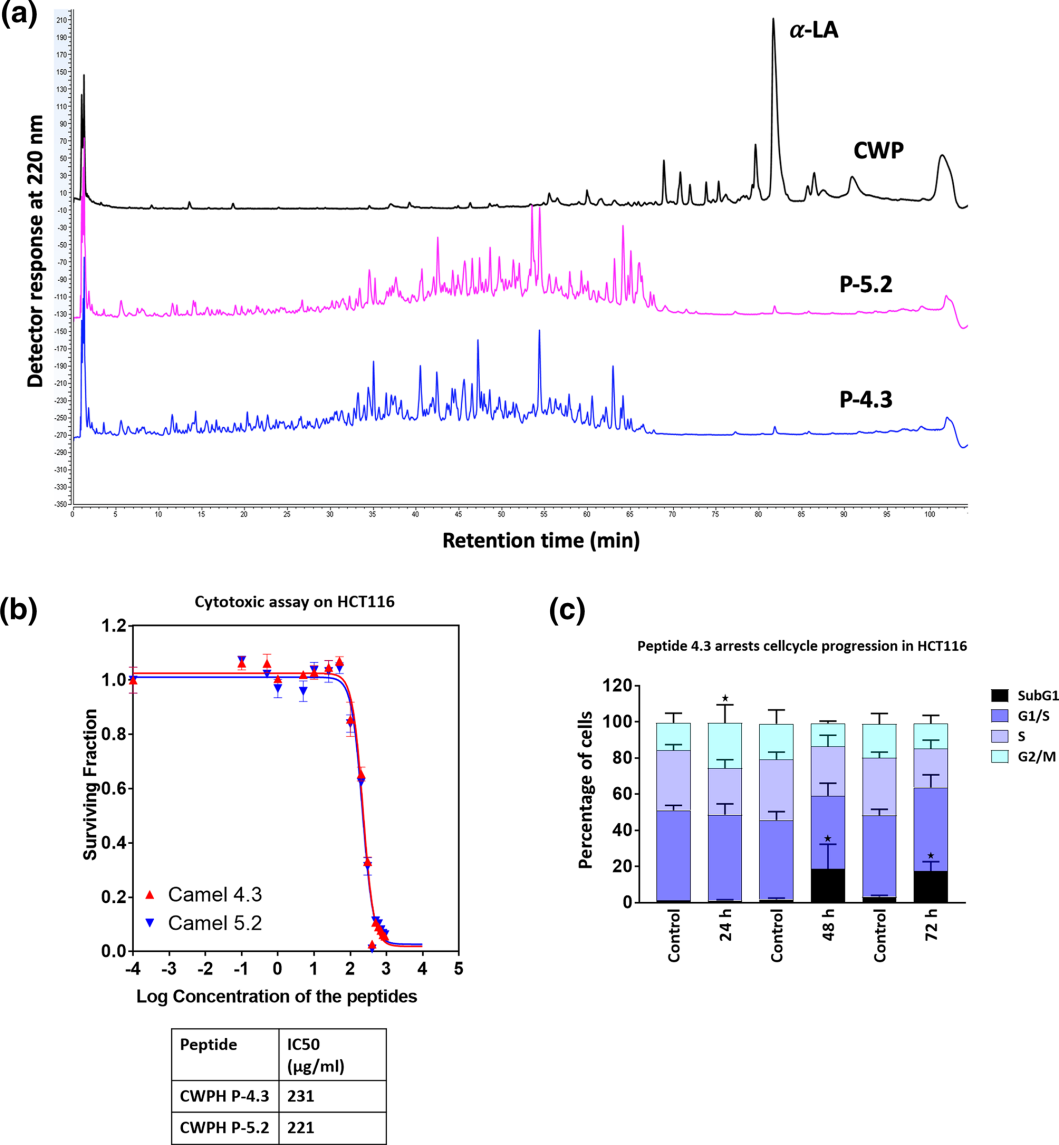
Peptide profiling and anti-proliferative screening of camel whey protein hydrolysates (P-4.3 and P-5.2). (**a**) RP-UPLC peptide profile of CWP and their pepsin generated hydrolysates P-5.2 and P-4.3. (**b**) Camel whey protein hydrolysates P-4.3 and P-5.2 inhibit growth of HCT116 cells. Viability of HCT116 cells after treatment with increasing concentrations of the CWPHs P-4.3 and P-5.2 for a period of 48 h. 231 μg/ml is the IC_50_ for CWPH P-4.3, 221 μg/ml is the IC_50_ for CWPH P-5.2. (**c**) Quantitative distribution of HCT116 cells in different phases of the cell cycle after treatment with camel CWPH P-4.3 (231 μg/ml) over a period of 24 h, 48 h and 72 h. Statistical analysis was carried out by student’s t-test using GraphPad Prism software and p < 0.05 was considered as statistically significant. *p < 0.05, **p < 0.01, ***p < 0.001 and ****p < 0.0001.

Reverse phase-ultra performance liquid chromatography (RP-UPLC) was used for the characterization of intact CWP and its peptic hydrolysates. The results obtained are presented in Fig. 1a. It was revealed that for intact CWP, a single sharp peak belonging to α-lactalbumin (LA) was observed between 80 and 85 min. Within the retention time ranging between 70–80 min, other eluted peaks mostly representing other minor whey proteins such as serum globulins, lactoferrin, immunoglobulin, lactoperoxidase were observed. Pepsin hydrolysis led to complete degradation of all the intact whey proteins as indicated by the peptide profile (Fig. 1a). The results are consistent with previous findings where simulated gastrointestinal digestion of camel whey protein concentrates treated with pepsin, trypsin and chymotrypsin resulted in complete hydrolysis of camel whey proteins^1^. The results indicate that camel whey protein hydrolysates contain a significant proportion of medium size peptides spanning over a wide range of hydrophobicity and eluted between 35 and 70 min. Smaller peaks of shorter peptides were also observed between the retention time 0–30 min. Previous studies have also reported that different camel milk protein hydrolysates generated with trypsin displayed short peptide peaks which were eluted over a range of retention time, indicating that camel milk protein hydrolysates contained peptides having a wide range of hydrophobicity^15^.

### Effects of hydrolysis parameters (E:S, time and temperature) on anti-inflammatory activities

Inflammation is one of the most valuable mechanisms for defence or life-threatening side effects in our body. However, secretion of enormous amounts of inflammatory mediators including prostaglandin-E2 (PGE2), nitric oxide (NO), and pro-inflammatory cytokines such as IL-1β, TNF-α and IL-6 t has been associated with diseases like obesity, diabetes and cancer. The search for anti-inflammatory food-based bioactive compounds has shown that milk and its derived bioactive peptides can potentially be used as potent anti-inflammatory food derived bioactive compounds^16^. The anti-inflammatory activities of CWPHs are presented as equivalent to a standard anti-inflammatory drug diclofenac sodium per mg of protein equivalent [DSE (µg/mg PE)] in Table 1. Results suggest that the intact camel whey proteins displayed inherent anti-inflammatory capacity. Similar effect was reported in streptozotocin-induced diabetic rats supplemented with camel undenatured whey protein^17^. The hydrolysis of CWP with pepsin demonstrated a significant increase (p < 0.05) in the anti-inflammatory effect of hydrolysates as compared to intact CWP (Table 1). Overall, the anti-inflammatory activity of 18 hydrolysates vary between 732.50 (P6.1) and 3779.16 (P2.1) µg DSE/mg PE. Some of the hydrolysates generated herein with camel whey exhibited activity higher than those reported earlier^18^, whereas others were comparable to those reported by Kamal et al.^9^. Previous studies have reported the role of milk derived bioactive peptides on the reduction of inflammation and an anti-inflammatory peptide “VPP” was identified^19^. Furthermore, alcalase and thermolysis driven hydrolysis of zein protein has been reported to produce three novel peptides PPYLSP, IIGGAL, and FLPPVTSMG with strong anti-inflammatory activities that regulate the expression of inflammation marker VCAM and ICAM^20^. The present study conclusively establishes the anti-inflammatory potential of camel whey protein derived hydrolysates and pave a way for further exploration to elucidate the molecular mechanism for anti-inflammatory properties of those hydrolysates.

### Cytotoxicity of selected CWPHs against HCT116 cancer cells

Anti-proliferative effects of milk proteins have been studied earlier and are emerging as highly promising agents to overcome the relapse and side effects of chemotherapy^21^. Camel milk is known to induce growth arrest in breast cancer cells^22^ and trigger significant apoptosis effect in HepG2 cells^23^. 18 CWPHs were screened for anti-cancer activity on HepG2 cells (Supplementary Fig. S1) and two promising CWPHs P-4.3 and P-5.2 on HCT116 cells (Fig. 1b). HCT116 cells turned out to be a better model for studying the anti-oncogenic mechansism. The effect of CWPHs on the survival of HCT116 cells was investigated both at low (0.01–10 μg/ml) and high (10–1000 μg/ml) concentration ranges. Sigmoidal concentration–response curve fitting showed that the concentration needed to reduce the survival to 50% (IC_50_) for the CWPH P-4.3 and P5.2 was 231.0 and 221.0 μg/ml, respectively.

### Analysis of cell cycle distribution induced by CWPHs

Effect on cell cycle distribution by CWPH P-4.3 and P5.2 on HCT116 was investigated by FACS analysis. In cancer, therapeutic molecules disrupt the natural balance of cancer cells either by modulating cell cycle check points or eliciting unrepairable DNA damage and eventually promoting cell death. Earlier study where milk proteins induced cell cycle arrests^24^ encouraged us to investigate the molecular machinery of these hydrolysates. P-4.3 (231 μg/ml) showed a significant (p < 0.05) G2/M arrest when treated for 24 h (15–25%), 48 h (2–19%) and 72 h (3–17%) (Fig. 1c). This could indicate that the P-4.3 induced apoptosis of the HCT116 cells or triggered non-cytotoxic growth arrest. P-5.2 (221 μg/ml) did not show any significant and consistent effect on the cell cycle progression of HCT116 cells. Hence, CWPH P-4.3 was further subjected to molecular analysis of its effector molecules.

### Effect of CWPH P-4.3 on cell cycle variables

**Figure 2:**
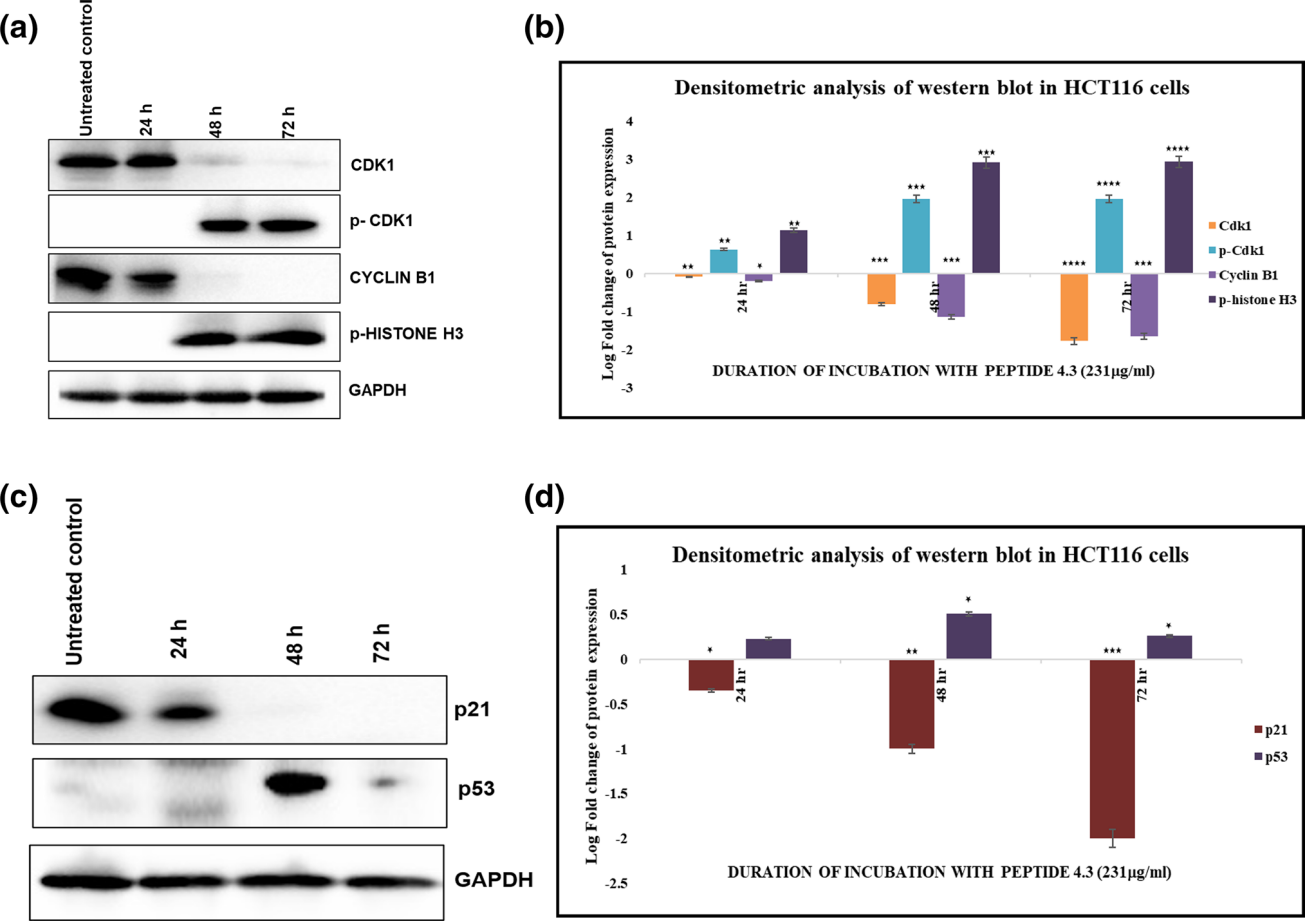
Inhibitory effect of CWPH P-4.3 on cell cycle progressive markers in HCT116 cells. (**a**, **c**) Western blot analysis of cell cycle regulatory proteins from HCT116 treated with CWPH P-4.3 (231 μg/ml) over a period of 24 h, 48 h and 72 h. (**b**, **d**) Each band intensity was quantified to analyze the protein expression using ImageJ, normalized relative to their respective loading control bands. Values are expressed as ratio of untreated control in log fold. Statistical analysis was carried out by student’s t-test using GraphPad Prism software and p < 0.05 was considered as statistically significant. *p < 0.05, **p < 0.01, ***p < 0.001 and ****p < 0.0001.

CWPH P-4.3-induced G2/M arrest in HCT116. The protein expression analyses of HCT116 with or without P-4.3 treated (231 μg/ml at 24 h, 48 h & 72 h) were done using western blot (Fig. 2). Cyclin dependent kinase 1(Cdk1) normally binds to Cyclin B1 to form a major regulator of mitosis. The synthesis and nuclear import of activated cyclin B1/Cdk1 complex peaks in late S phase and early G2 phase. Inactivation of Cdk1 and proteolysis of Cyclin B1 halts cell division and promotes cells to exit mitosis. In the present study, CWPH P-4.3 decreased the expression of Cdk1 and cyclin B1 in a time-dependent manner (Fig. 2a,b) translating the G2/M arrest^25^. In cell cycle, each check points are tightly regulated to maintain the genomic integrity and cellular homeostasis. The inhibitory phosphorylation of Cdk1 at Tyr15 and Thr14 is a feedback mechanism that is known to regulate cell cycle. It is conjectured that the DNA damage occurred at G2 phase of cell cycle, sustains Cdk1 in a Thr14- and Tyr15-phosphorylated inactive state^26^. Here, there was a significant induction in the p-Cdk1(Tyr15) expression confirming the regulatory effect which resulted in cell cycle arrest (Fig. 2a,b). Phosphorylation of histone H3 Ser-10 (p-HH3) is known to take place when cells are treated with antiproliferative agents indicating DNA damage^27^. FACS illustrated a subG1 phase at 48 and 72 h post treatment (Fig. 1c) and that was significantly mirrored in the protein levels of p-HH3 (Fig. 2a,b) proving a DNA damage induced by the P-4.3^28^.

We investigated the effect of p53/p21 cross talk post P-4.3 treatment. p53 is a tumour suppressor that acts as a dominant node in the cellular pathways responding to various events like DNA damage, oncogenic stimuli and chemical induced stress. Overexpression of p53 has been reported to induce both growth arrest and apoptosis^29^. The CDK inhibitor p21 (also known as p21WAF1/Cip1) acts as a negative regulator of p53-dependent apoptosis. P21 inactivates cyclinB1/Cdk1 complex leading to cell cycle arrest^30^. In this study, at 48 h following the CWPH P-4.3 treatment, an induction of p53 and a p21 inhibition were observed (Fig. 2c,d). Interruptions of tightly regulated pathways are classic properties of cancer cells backing the irregularity in the p21/p53 pathway when exposed to our antiproliferative agent. Protein expression of p21 is at variance with that of p53 amongst the treated and untreated HCT116 cells. This may indicate a p53 independent mechanism by the CWPH. A variety of transcription factors have proven to regulate p21 independent of p53 and p150^Sal2^, is an example^31^. This opens doors to more studies in order to understand the CWPH induced anti-cancer effect.

### Identification and physicochemical properties of peptide

Based on their significant potential anticancer effect (Fig. 1b), CWPHs P-4.3 and P-5.2 were processed for peptide sequence identification and the interaction of selected peptides with target protein via in-silico analysis was also carried out. All results relevant to the number of peptides, their sequence, physicochemical properties and predictions related to their anti-inflammatory and anti-cancer potential are presented in Table 2. Overall, 162 and 44 peptides were identified in CWPH P-4.3 and P-5.2, respectively (Supplementary material—Table S1). Depending on the average local confidence (ALC) value of at least 80% and on the peptide ranker score of > 0.50, peptides were represented as potentially biologically active peptides (BAPs). A total of 42 and 19 peptides from P-4.3 and P-5.2, respectively were qualified (peptide ranker score of > 0.5) to be BAPs (Table 2). The molecular weight of these BAPs varied from 234.32 g/mol for LC (P-5.2) to 1563.79 g/mol for AVLPFQEPVPDPVR (P-4.3). Isoelectric point values varied between pH 0.9 and 10.9 among generated peptides. It has been previously reported that the anti-inflammatory action of proteins or their derived peptides is highly dependent on the sequence and structure of the peptides. Buffalo milk casein-derived decapeptide suppressed inflammatory cytokine levels, splenocytes proliferation and improved the phagocytosis in mice which are the classic anti-inflammatory indicators^32^. αS1-casein derived peptide has been shown to inhibit the matrix metalloproteinases (MMP-9) activity in HT-29 and SW480 cells^33^. Hence, anti-cancer peptide (ANTICP) and anti-inflammatory (AIP) web-based prediction servers, were used to determine the anticancer and anti-inflammatory peptides, respectively. They utilize support vector machine (SVM) models developed based on amino acid composition and binary profile features inorder to predict the activity of the peptides. All peptides except DVTVLDNTDGK (P-4.3), ESLPGVPPPSGQPLL and DLPLMT (P-5.2) were predicted to be anti-inflammatory (AIP). Toxicity impedes the inclusion of BAPs in the food and pharmaceutical industry. Therefore, resulted peptides were screened for their toxicity using ToxinPred and none of them turned out to be toxic. In addition, all peptides except GLFQ, GLHPVPQP and GLHPVPQPLV (P-4.3) were predicted to have anti-cancer potential.

**Table 2:** In silico analysis of anti-inflammatory, anti-cancer and toxicity prediction of identified potential biologically active peptides from CWPHs P4.3 and 5.2 as revealed by their peptide ranker score, ToxinPred, Anticp and AIPpred.

Peptide sequence	Peptide ranker score	Mol wt (g/mol)	Charge	Isoelectric point	Toxicity	AIP prediction	Anticp prediction
**P-4.3**							
AHLEQVLLR	0.83	1078.27	0.1	7.88	Non-toxin	AIP	Anticp
ALPNIDPPTVER	0.82	1321.48	− 1	3.93	Non-toxin	AIP	Anticp
AVLPFQEPVPDPVR	0.75	1563.79	− 1	3.93	Non-toxin	AIP	Anticp
AVVSPIQF	0.75	860.01	0	3.77	Non-toxin	AIP	Anticp
AVVSPIQFR	0.75	1016.19	1	10.9	Non-toxin	AIP	Anticp
DDVVIK	0.74	687.78	− 1	3.71	Non-toxin	AIP	Anticp
DFLK	0.74	521.61	0	6.66	Non-toxin	AIP	Anticp
DILK	0.74	487.59	0	6.66	Non-toxin	AIP	Anticp
DILKEDMPSQR	0.69	1331.5	− 1	4.17	Non-toxin	AIP	Anticp
DLENLHLPLPLL	0.68	1386.63	− 1.9	3.92	Non-toxin	AIP	Anticp
DLENLHLPLPLLQ	0.68	1514.76	− 1.9	3.92	Non-toxin	AIP	Anticp
DNENLQSR	0.66	974.97	− 1	3.93	Non-toxin	AIP	Anticp
DSALGLLR	0.65	843.97	0	6.68	Non-toxin	AIP	Anticp
DV	0.65	232.23	− 1	0.72	Non-toxin	AIP	Anticp
DVQPTLSPGDR	0.63	1184.26	− 1	3.71	Non-toxin	AIP	Anticp
DVTVLDNTDGK	0.62	1176.23	− 2	3.41	Non-toxin	Non-AIP	Anticp
EDLVSK	0.62	689.75	− 1	3.93	Non-toxin	AIP	Anticp
EDLVSKDDVVIK	0.61	1359.52	− 2	3.82	Non-toxin	AIP	Anticp
EDLVSKDDVVIKS	0.6	1446.6	− 2	3.82	Non-toxin	AIP	Anticp
ELAVVSPIQFR	0.59	1258.47	0	6.86	Non-toxin	AIP	Anticp
ELTPGAATTLEGK	0.59	1287.42	− 1	4.15	Non-toxin	AIP	Anticp
ENIDELKDTR	0.58	1232.3	− 2	3.93	Non-toxin	AIP	Anticp
ENLHLPLPLL	0.58	1158.39	− 0.9	5.1	Non-toxin	AIP	Anticp
ENLHLPLPLLQ	0.58	1286.52	− 0.9	5.1	Non-toxin	AIP	Anticp
EPIPYPILP	0.58	1038.24	− 1	1.1	Non-toxin	AIP	Anticp
EPVPDPVR	0.57	908.01	− 1	3.93	Non-toxin	AIP	Anticp
EPVPDPVRG	0.57	965.06	− 1	3.93	Non-toxin	AIP	Anticp
ETAAEVELR	0.56	1017.09	− 2	3.85	Non-toxin	AIP	Anticp
ETIIPK	0.56	699.84	0	6.85	Non-toxin	AIP	Anticp
ETMDFLK	0.55	883.02	− 1	3.93	Non-toxin	AIP	Anticp
FE	0.54	294.3	− 1	pH 1	Non-toxin	AIP	Anticp
FLDDDLTDDK	0.54	1196.22	− 4	3.11	Non-toxin	AIP	Anticp
FLPPLQPAV	0.53	981.19	0	3.41	Non-toxin	AIP	Anticp
FLPPLQPAVM	0.52	1112.39	0	3.46	Non-toxin	AIP	Anticp
FPHASEVVKPQ	0.52	1238.39	0.1	7.56	Non-toxin	AIP	Anticp
FQEPVPDPVR	0.52	1183.31	− 1	3.93	Non-toxin	AIP	Anticp
FRQENIDELK	0.51	1291.41	− 1	4.32	Non-toxin	AIP	Anticp
FRQENIDELKDTR	0.51	1663.79	− 1	4.44	Non-toxin	AIP	Anticp
GAHAGPTWNPISI	0.51	1320.45	0.1	7.81	Non-toxin	AIP	Anticp
GLFQ	0.51	463.53	0	3.7	Non-toxin	AIP	Non-Anticp
GLHPVPQP	0.5	843.97	0.1	7.81	Non-toxin	AIP	Non-Anticp
GLHPVPQPLV	0.5	1056.26	0.1	7.81	Non-toxin	AIP	Non-Anticp
**P-5.2**							
LRPFL	0.93	644.81	1	0.84	Non-toxin	AIP	Anticp
LRFPL	0.91	644.81	1	0.84	Non-toxin	AIP	Anticp
LC	0.84	234.32	− 0.1	3.11	Non-toxin	AIP	Anticp
HSGF	0.81	446.46	0.1	7.56	Non-toxin	AIP	Anticp
LWKL	0.79	558.71	1	0.12	Non-toxin	AIP	Anticp
PLLL	0.74	454.6	0	4.08	Non-toxin	AIP	Anticp
ESLPGVPPPSGQPLL	0.74	1487.7	− 1	0.92	Non-toxin	Non-AIP	Anticp
KPVMECGALLL	0.7	1173.49	− 0.1	6.12	Non-toxin	AIP	Anticp
CHLL	0.68	484.62	0	7.09	Non-toxin	AIP	Anticp
CFNTLMLPPEPLL	0.67	1487.83	− 1.1	0.92	Non-toxin	AIP	Anticp
SHGM	0.66	430.48	0.1	7.54	Non-toxin	AIP	Anticp
QVNLPLTGGML	0.64	1142.37	0	3.34	Non-toxin	AIP	Anticp
HSYAEEPPLYPLL	0.63	1528.7	− 1.9	4.14	Non-toxin	AIP	Anticp
DLPLMT	0.6	688.84	− 1	0.75	Non-toxin	Non-AIP	Anticp
NNLPLTM	0.58	801.95	0	3.29	Non-toxin	AIP	Anticp
PLMLPLHQPL	0.57	1158.46	0.1	8.26	Non-toxin	AIP	Anticp
DLLPLTM	0.57	801.99	− 1	0.78	Non-toxin	AIP	Anticp
FLEL	0.55	520.62	− 1	0.92	Non-toxin	AIP	Anticp
NHCDEPVPMT	0.54	1142.27	− 2	3.92	Non-toxin	AIP	Anticp

*Anticp* anti-cancer peptide, *AIP* anti-inflammatory peptide.

### Macromolecular target prediction

An in silico approach of molecular docking was done using two peptide sequences (AHLEQVLLR and ALPNIDPPTVER) derived from camel whey protein hydrolysate P-4.3 (score ˃ 0.8) (Table 2), in an attempt to search for its most probable macromolecular protein targets. For this purpose, we initiated this by utilizing a number of web-based search engines. The first engine being utilized was the SwissTargetPrediction server, where it predicts bioactivity based on 2D/3D similarity search of 370 K active compounds spaning over 3 K macromolecular protein targets. Moreover, MolTarPred search engine was utilized to scan over 600 K active compounds and over 4.5 K macromolecular targets, while the PASS server was utilized to scan more than 260 K compounds with known biological activities employing a knowledge base-rules about their structure–activity relationships. On the other hand, cross-target similarity maps were predicted via the SEA (Similarity Ensemble Approach) search engine that produces its results based on chemical similarity and biological clustering. The results obtained from these web-based search engines were further filtered based on their top-ranked scores and oncogenic target involvement. Finally, the macromolecular targets involvement hypotheses were judged based on their initial biological screening (i.e., western-blot). Accordingly, a number of biological targets were nominated for the initial biological screening, nevertheless one target namely; PLK-1 Polo box domain was confirm and supported by the biological data.

**Figure 3:**
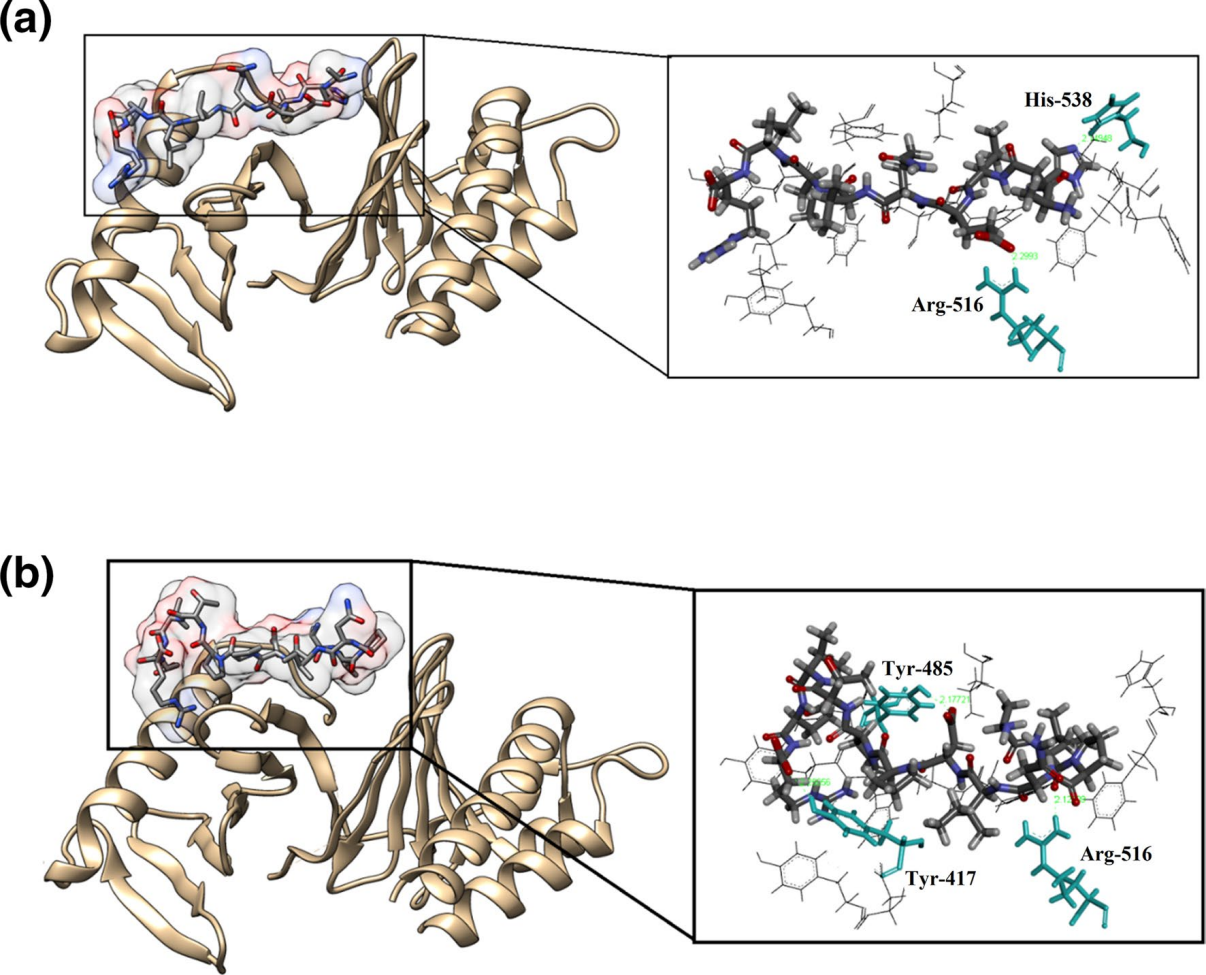
Investigated peptides binding modes within the active site of PLK-1 Polo box domain (PDB-ID: 3RQ7). (**a**) Binding mode of peptide-1 (AHLEQVLLR) from CWPHs P-4.3 within the active site of PLK-1, (**b**) Binding mode of peptide-2 (ALPNIDPPTVER) from CWPHs P-4.3 within the active site of PLK-1. Left panels illustrated the overall binding site, while the right panels illustrated hydrogen bond interactions with the residues of the active site. Ligands and important amino acids residues are presented in stick rendering, while H-bond interactions are shown as green dashed lines with their corresponding distances in angstrom.

The most probable binding modes for peptides within the Polo like kinase 1 (PLK1) were explored by performing molecular docking calculations and employing MDockPeP server. For peptide-1 (AHLEQVLLR), the model with the highest ITScorePeP (− 160.441) was considered. Peptide-1 (AHLEQVLLR) demonstrated effective binding efficiency with the enzyme active site (Fig. 3a left) and was able to establish two hydrogen bond interactions with the corresponding Arg-516 and His-538 amino acid residues (Fig. 3a right). On the other hand, the binding mode of peptide-2 (ALPNIDPPTVER) was estimated to be more significant with a calculated ITScorePeP of − 162.385. The binding mode of Peptide-2 was in agreement with that of peptide-1 (Fig. 3b left), and peptide-2 was able to establish three hydrogen bond interactions with the Tyr-417, Tyr-485 and Arg-516 active site residues (Fig. 3b right). A strong interactive profile was found among Polo-like kinase-1 (Plk1) with both the peptide1 and 2 (Fig. 3a,b).

These docking analysis results are consistent with our in vitro analysis where Plk1 expression is crucial for controlling G2/M phase of the cell cycle and is overexpressed in many tumour types. In pre-clinical studies, Plk1 inhibitors were showing significant upregulation in the expression of the mitotic marker p-Histone H3. PLK1 induced phosphorylation of cyclin B1 is necessary for the formation of Cdk1/Cyclin B1 complex which in turn initiates the mitotic entry of a cell^34^. There are two projected models demonstrating PBD mediated PLK1 kinase activity. Our data suggests a ‘Processive phosphorylation’ model keeping into account of the CDK1 phosphorykation^35^. Taken together, Plk1 seems to have a major role in inducing the anti-mitotic and pro-apoptotic effects imparted in vitro by the camel whey derived bioactive peptides.

### In silico structural activity relationship (SAR) of identified peptides to Polo-Like Kinase 1 (PLK1)

Active site interaction between PLK-1 (PDB code: 3RQ7) and bioactive peptides from CWPHs (P-4.3 and P-5.2) were studied using PepSite 2. Human protein Polo-Like Kinase 1 (PLK1) plays an important regulatory role in mitosis and cellular proliferation through phosphorylation of different substrates. PLK1 is found to be overexpressed in a variety of cancers like prostrate and colorectal cancers^36^. Therefore, potential inhibitors of this protein are sought after for developing novel anti-cancer drugs. Inhibiting the activity of PLK1 through phospho-ligand binding of the Polo Box Domain (PBD) is a potential cancer therapy as it might prevent the completion of the cell cycle and induce apoptosis. Therefore, an intriguing array of novel PLK1 inhibitors is being explored for their potential inhibitory activity against PLK1. The detailed structural analysis of PLK1 has revealed that the PBD is important for PLK1 activity. It was reported that Val415, Tyr417, Tyr421, Leu478, Tyr481, Phe482 and Tyr485 are recognised as the cryptic binding pockets on the PLK-1 PBD surface^37^. These residues regulate the substrate recognition by adopting open and closed conformations and by binding a peptide at these PBD surface residues, substrate discrimation could occur, thus inhibiting the overexpression of PLK1 in the tumor cells. Table 3 shows the *p* value as a measure to binding strength, reactive residue in the peptides and the potential binding sites of the identified peptides with PLK-1. It was predicted that most of the peptides in P-4.3 and P-5.2 hydrolysates were capable of binding to 5–7 cryptic binding sites (CBS) of PLK1. These CBS binding sites essentially mirror the anticancer properties of CWPH P-4.3.

**Table 3:** The Pepsite 2 p-values, reactive residues and potential binding sites of the selected peptides within the active site of PLK-1 Polo box domain (PDB-ID: 3RQ7).

Peptide	Pepsite-2 p-value	Reactive peptide residues	Bound sites (3RQ7)	CBS
**P-4.3**				
AHLEQVLLR	0.02195	A1, H2, L3, E4, Q5, V6	Val415^a^, Tyr417^a^, Tyr481^a^, Phe482^a^, Tyr485^a^, His489	5
ALPNIDPPTVER	0.001197	A1, L2 P3, N4, I5, P7, P8, T9, V10, R12	Val415^a^, Tyr417^a^, Tyr421^a^, Lys474, Leu478^a^, Tyr481^a^, Phe482^a^, Tyr485^a^, His489	7
AVLPFQEPVPDPVR	0.0008096	A1, V2, L3, P4, F5, Q6, P8, P10, D11, P12, V13, R14	Val415^a^, Tyr417^a^, Tyr421^a^, Lys474, Leu478^a^, Tyr481^a^, Phe482^a^, Tyr485^a^, His489	7
AVVSPIQF	0.003612	V2, S3, P5, I6, Q7, F8	Tyr417^a^, Lys420, Tyr421^a^, Leu478^a^, Tyr481^a^, Phe482^a^, Tyr485^a^, His489	6
AVVSPIQFR	0.004457	V3, P5, I6, Q7, F8, R9	Tyr417^a^, Lys420, Tyr421^a^, Lys474, Leu478^a^, Tyr481^a^, Phe482^a^, Tyr485^a^	6
DDVVIK	0.01484	D1, V3, V4, I5, K6	Val415^a^, Tyr417^a^, Tyr421^a^, Leu478^a^, Tyr481^a^, Phe482^a^, Tyr485^a^	7
DFLK	0.01728	D1, F2, L3, K4	Val415^a^, Tyr417^a^, Tyr421^a^, Lys474, Leu478^a^, Tyr481^a^, Phe482^a^, Tyr485 ^a^	7
DILK	0.01554	D1, I2, L3, K4	Tyr417^a^, Tyr421^a^, Lys474, Leu478^a^, Tyr481^a^, Phe482^a^, Tyr485 ^a^	6
DILKEDMPSQR	0.01104	D1, I2, L3, K4, D6, M7, P8, Q10, R11	Val415^a^, Tyr417^a^, Tyr421^a^, Lys474, Leu478^a^, Tyr481^a^, Phe482^a^, Tyr485 ^a^	7
DLENLHLPLPLL	0.01863	D1, E3, N4, L5, H6, L7, P8, L9, P10, L12	Val415^a^, Tyr417^a^, Lys420, Tyr421^a^, Leu478^a^, Tyr481^a^, Phe482^a^, Tyr485 ^a^	7
DLENLHLPLPLLQ	0.01821	D1, E3, N4, L5, H6, L7, P8, L9, P10, L12, Q13	Val415^a^, Tyr417^a^, Lys420, Tyr421^a^, Leu478^a^, Tyr481^a^, Phe482^a^, Tyr485^a^, His489	7
DNENLQSR	0.007954	D1, N2, E3, N4, L5, Q6	Val415^a^, Tyr417^a^, Tyr421^a^, Leu478^a^, Tyr481^a^, Phe482^a^, Tyr485 ^a^	7
DSALGLLR	0.01869	S2, A3, L4, G5, L6, R7	Val415^a^, Tyr417^a^, Tyr421^a^, Lys474, Leu478^a^, Tyr481^a^, Phe482^a^, Tyr485^a^, His489	7
DV	0.09958	D1, V2	Tyr417^a^, Leu478^a^, Tyr481^a^, Phe482^a^, Tyr485 ^a^	5
DVQPTLSPGDR	0.006228	V2, Q3, P4, T5, L6, P8, G9, D10, R11	Val415^a^, Tyr417^a^, Lys420, Tyr421^a^, Gly422, Lys474, Leu478^a^, Tyr481^a^, Phe482^a^, Tyr485 ^a^	7
DVTVLDNTDGK	0.02695	D1, T3, V4, L5, D6, N7, T8, G10, K11	Val415^a^, Tyr417^a^, Tyr421^a^, Lys474, Leu478^a^, Tyr481^a^, Phe482^a^, Tyr485 ^a^	7
EDLVSK	0.06053	D2, L3, V4, S5, K6	Tyr417^a^, Tyr421^a^, Lys474, Leu478^a^, Tyr481^a^, Phe482^a^, Tyr485^a^, His489	6
EDLVSKDDVVIK	0.03769	D2, L3, V4, S5, K6, D7, V9, V10, I11, K12	Val415^a^, Tyr417^a^, Tyr421^a^, Lys474, Leu478^a^, Tyr481^a^, Phe482^a^, Tyr485^a^, His489	7
EDLVSKDDVVIKS	0.03637	D2, L3, V4, S5, K6, D7, D8, V9, V10, I11, K12	Tyr417^a^, Tyr421^a^, Lys474, Leu478^a^, Tyr481^a^, Phe482^a^, Tyr485^a^, His489	6
ELAVVSPIQFR	0.01091	L2, A3, V4, V5, S6, P7, I8, Q9, F10	Tyr417^a^, Tyr421^a^, Lys474, Leu478^a^, Tyr481^a^, Phe482^a^, Tyr485^a^, His489	6
ELTPGAATTLEGK	0.01091	E1, L2, T3, P4, A6, A7, T8, T9, L10, E11, K13	Val415^a^, Tyr417^a^, Tyr421^a^, Lys474, Leu478^a^, Tyr481^a^, Phe482^a^, Tyr485^a^, His489	7
ENIDELKDTR	0.04065	N2, I3, L6, D8, T9, R10	Tyr417^a^, Lys420, Tyr421^a^, Lys474, Leu478^a^, Tyr481^a^, Phe482^a^, Tyr485^a^, His489	6
ENLHLPLPLL	0.004611	H4, L5, P6, L7, P8, L10	Val415^a^, Tyr417^a^, Lys420, Tyr421^a^, Lys474, Leu478^a^, Tyr481^a^, Phe482^a^, Tyr485^a^, His489	7
ENLHLPLPLLQ	0.01709	N2, L3, H4, L5, P6, L7, P8, L10, Q11	Val415^a^, Tyr417^a^, Tyr421^a^, Lys474, Leu478^a^, Tyr481^a^, Phe482^a^, Tyr485^a^, His489	7
EPIPYPILP	0.001003	I3, P4, P6, I7, L8, P9	Val415^a^, Tyr417^a^, Lys420, Tyr421^a^, Lys474, Leu478^a^, Tyr481^a^, Phe482^a^, Tyr485 ^a^	7
EPVPDPVR	0.001355	P2, P4, D5, P6, V7, R8	Val415^a^, Tyr417^a^, Tyr421^a^, Lys474, Leu478^a^, Tyr481^a^, Phe482^a^, Tyr485^a^, His489	7
EPVPDPVRG	0.001965	P2, P4, D5, P6, V7, R8	Val415^a^, Tyr417^a^, Tyr421^a^, Lys474, Leu478^a^, Tyr481^a^, Phe482^a^, Tyr485^a^, His489	7
ETAAEVELR	0.02771	T2, A3, A4, V6, L8, R9	Val415^a^, Tyr417^a^, Tyr421^a^, Lys474, Leu478^a^, Tyr481^a^, Phe482^a^, Tyr485^a^, His489	7
ETIIPK	0.004608	T2, I3, I4, P5, K6	Val415^a^, Tyr417^a^, Tyr421^a^, Lys474, Leu478^a^, Tyr481^a^, Phe482^a^, Tyr485 ^a^	7
ETMDFLK	0.007162	T2, M3, D4, F5, L6, K7	Val415^a^, Tyr417^a^, Tyr421^a^, Lys474, Leu478^a^, Tyr481^a^, Phe482^a^, Tyr485^a^, His489	7
FE	0.07216	F1, E2	Tyr417^a^, Tyr421^a^, Tyr485 ^a^	3
FLDDDLTDDK	0.04962	D3, D4, L6, T7, D8, D9	Tyr417^a^, Tyr421^a^, Leu478^a^, Tyr481^a^, Phe482^a^, Tyr485 ^a^	6
FLPPLQPAV	0.0006121	F1, P3, P4, L5, Q6, P7	Tyr417^a^, Tyr421^a^, Lys 474, Leu478^a^, Tyr481^a^, Phe482^a^, Tyr485^a^, His489	6
FLPPLQPAVM	0.0008342	F1, P3, P4, L5, Q6, P7	Tyr417^a^, Tyr421^a^, Lys 474, Leu478^a^, Tyr481^a^, Phe482^a^, Tyr485^a^, His489	6
FPHASEVVKPQ	0.00126	F1, P2, H3, A4, S5, V8, K9, P10, Q11	Val415^a^, Tyr417^a^, Lys420, Tyr421^a^, Leu478^a^, Tyr481^a^, Phe482^a^, Tyr485^a^, His489	7
FQEPVPDPVR	0.001478	Q2, E3, P4, V5, P6, P8	Val415^a^, Tyr417^a^, Tyr421^a^, Lys 474, Leu478^a^, Tyr481^a^, Phe482^a^, Tyr485^a^, His489	7
FRQENIDELK	0.01164	F1, R2, Q3, N5, I6, D7	Val415^a^, Tyr417^a^, Lys420, Tyr421^a^, Lys474, Leu478^a^, Tyr481^a^, Phe482^a^, Tyr485 ^a^	7
FRQENIDELKDTR	0.0108	F1, R2, Q3, N5, I6, D7, L9, K10, D11, T12, R13	Val415^a^, Tyr417^a^, Lys420, Tyr421^a^, Gly422, Lys474, Leu478^a^, Tyr481^a^, Phe482^a^, Tyr485 ^a^	7
GAHAGPTWNPISI	0.007397	G1, H3, A4, P6, W8, N9, P10, I11, S12, I13	Val415^a^, Tyr417^a^, Lys420, Tyr421^a^, Lys474, Leu478^a^, Tyr481^a^, Phe482^a^, Tyr485^a^, His489	7
GLFQ	0.002078	G1, L2, F3, Q4	Val415^a^, Tyr417^a^, Tyr421^a^, Leu478^a^, Tyr481^a^, Phe482^a^, Tyr485 ^a^	7
GLHPVPQP	0.0002835	H3, P4, V5, P6, Q7, P8	Val415^a^, Tyr417^a^, Tyr421^a^, Lys474, Leu478^a^, Tyr481^a^, Phe482^a^, Tyr485^a^, His489	7
GLHPVPQPLV	0.0005692	H3, P4, V5, P6, Q7, P8	Val415^a^, Tyr417^a^, Tyr421^a^, Lys474, Leu478^a^, Tyr481^a^, Phe482^a^, Tyr485^a^, His489	7
**P-5.2**				
LRPFL	0.002388	R2, P3, F4, L5	Val415^a^, Tyr417^a^, Tyr421^a^, Lys474, Leu478^a^, Tyr481^a^, Phe482 ^a^	6
LRFPL	0.001516	R2, F3, P4, L5	Val415^a^, Tyr417^a^, Tyr421^a^, Leu478^a^, Tyr481^a^, Phe482^a^, Tyr485 ^a^	7
LC	0.02921	L1, C2	Tyr417^a^, Tyr421^a^, Lys474, Leu478^a^, Tyr481^a^, Phe482 ^a^	5
HSGF	0.01477	H1, S2, G3, F4	Val415^a^, Tyr417^a^, Tyr421^a^, Tyr481^a^, Phe482^a^, Tyr485^a^, His489	6
LWKL	0.01982	L1, W2, K3, L4	Tyr417^a^, Lys420, Tyr421^a^, Lys474, Leu478^a^, Tyr481^a^, Phe482^a^, Tyr485 ^a^	6
PLLL	0.00254	P1, L2, L3, L4	Val415^a^, Tyr417^a^, Lys420, Tyr421^a^, Lys474, Leu478^a^, Tyr481^a^, Phe482^a^, Tyr485 ^a^	7
ESLPGVPPPSGQPLL	0.007101	L3, P4, G5, V6, P7, P8, P9, S10, Q12, P13, L14, L15	Val415^a^, Tyr417^a^, Lys420, Tyr421^a^, Lys474, Leu478^a^, Tyr481^a^, Phe482^a^, Tyr485 ^a^	7
KPVMECGALLL	0.004389	K1, P2, V3, M4, C6, A8, L9, L10, L11	Val415^a^, Tyr417^a^, Tyr421^a^, Lys474, Leu478^a^, Tyr481^a^, Phe482^a^, Tyr485^a^, His489	7
CHLL	0.0129	C1, H2, L3, L4	Tyr417^a^, Tyr421^a^, Lys474, Leu478^a^, Tyr481^a^, Phe482^a^, Tyr485 ^a^	6
CFNTLMLPPEPLL	0.001375	C1, F2, N3, T4, M6, L7, P8, P9, E10, P11, L13	Val415^a^, Tyr417^a^, Lys420, Tyr421^a^, Lys474, Leu478^a^, Tyr481^a^, Phe482^a^, Tyr485^a^, His489	7
SHGM	0.004702	S1, H2, G3, M4	Val415^a^, Tyr417^a^, Tyr421^a^, Leu478^a^, Tyr481^a^, Phe482^a^, Tyr485^a^, His489	7
QVNLPLTGGML	0.009459	Q1, N3, L4, P5, L6, T1, G9, M10, L11	Val415^a^, Tyr417^a^, Lys420, Tyr421^a^, Leu478^a^, Tyr481^a^, Phe482^a^, Tyr485 ^a^	7
HSYAEEPPLYPLL	0.01899	H1, S2, Y3, A4, E6, P7, P8, L9, Y10, P11, L13	Val415^a^, Tyr417^a^, Lys420, Tyr421^a^, Lys474, Leu478^a^, Tyr481^a^, Phe482^a^, Tyr485^a^, His489	7
DLPLMT	0.002004	D1, L2, P3, L4, M5	Val415^a^, Tyr417^a^, Tyr421^a^, Lys474, Leu478^a^, Tyr481^a^, Phe482^a^, Tyr485^a^, His489	7
NNLPLTM	0.000809	N2, L3, P4, L5, T6, M7	Val415^a^, Tyr417^a^, Lys420, Tyr421^a^, Lys474, Leu478^a^, Tyr481^a^, Phe482^a^, Tyr485^a^, His489	7
PLMLPLHQPL	0.001916	M3, L4, P5, L6, H7, Q8	Val415^a^, Tyr417^a^, Lys420, Tyr421^a^, Lys474, Leu478^a^, Tyr481^a^, Phe482^a^, Tyr485^a^	7
DLLPLTM	0.001321	L2, L3, P4, L5, T6, M7	Val415^a^, Tyr417^a^, Tyr421^a^, Lys474, Leu478^a^, Tyr481^a^, Phe482^a^, Tyr485^a^, His489	7
FLEL	0.03925	F1, L2, E3, L4	Val415^a^, Tyr417^a^, Tyr421^a^, Leu478^a^, Tyr481^a^, Phe482^a^, Tyr485^a^	7
NHCDEPVPMT	0.004474	D4, P6, V7, P8, M9, T10	Val415^a^, Tyr417^a^, Lys420, Tyr421^a^, Lys474, Leu478^a^, Tyr481^a^, Phe482^a^	6

*CBS* no. of bound cryptic binding sites.

^a^Cryptic binding sites.

## Conclusion

**Figure 4:**
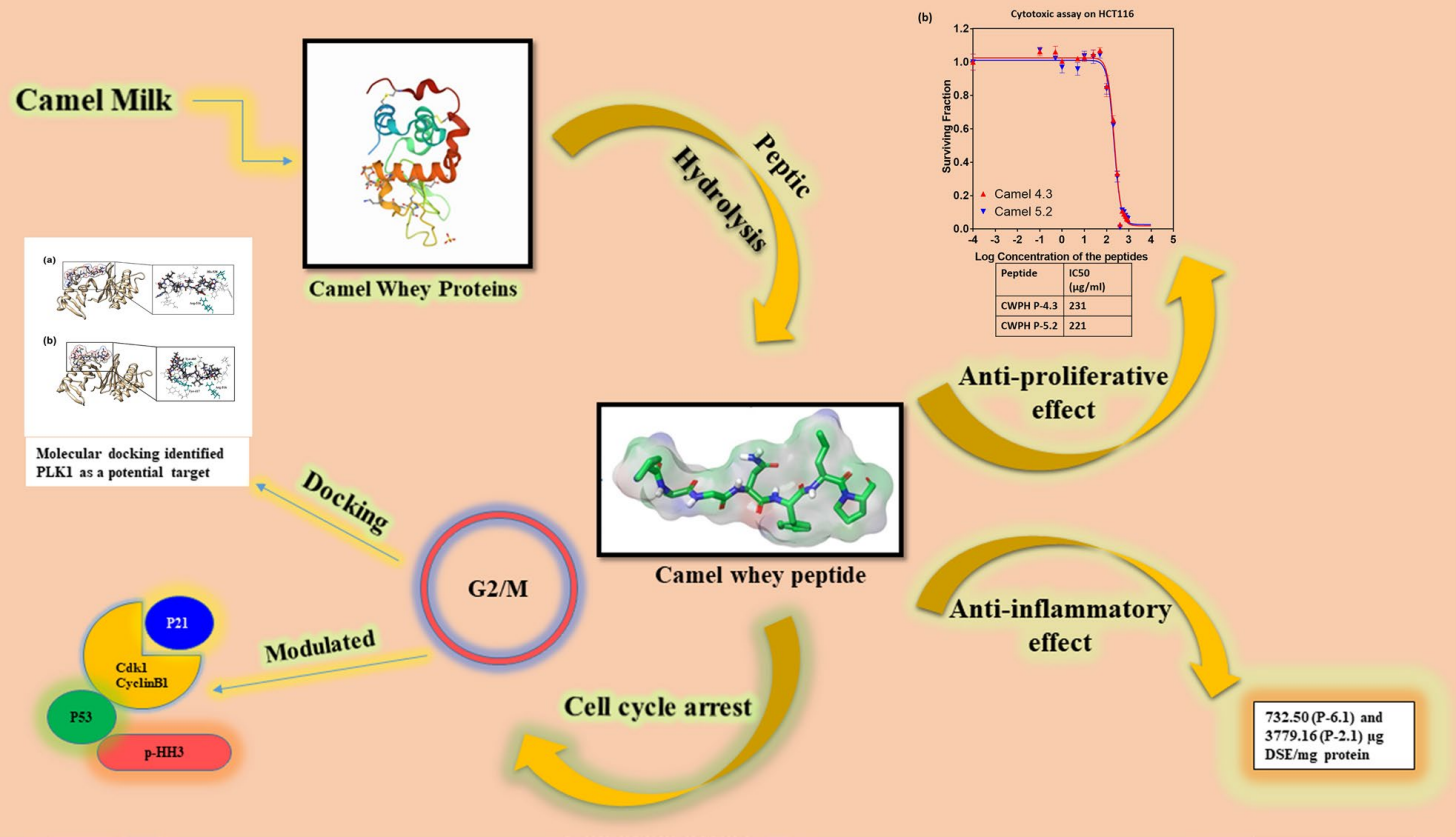
Camel whey protein hydrolysates induce G2/M cellcycle arrest in HCT116 cells. This graphical abstract shows the molecular mechanism employed by the camel whey protein hydrolysates in inducing anti-proliferative effect on the human colorectal cancer cells implicating PLK1 as a potential target.

The anti-cancerous properties of the pepsin digested CWPH have been demonstrated in this study for the first time divulging two promising peptide sequences AHLEQVLLR and ALPNIDPPTVER with a potential of target therapy. G2/M cell cycle markers were affected by the CWPH P-4.3. Data of the in-silico study also identifies PLKI-1 as a potential target of the CWPH P-4.3 (Fig. 4). Future studies on potential peptides sequence in a purified form should be carried out to investigate the anticancer activity in in-vitro as well as in-vivo model systems and their pharmacokinetic aspects like absorption, distribution, and bioavailability within the cells should be studied.

## Materials and methods

### Preparation of camel whey proteins

Camel whey proteins were prepared from raw camel milk samples obtained from healthy camels (*Camelus dromedaries*) kept at a local camel farm located in Al Ain, Abu Dhabi, United Arab Emirates (UAE). Camel milk samples were immediately transferred under refrigerated conditions to Food Chemistry laboratory at College of Food and Agriculture, United Arab Emirates University. The milk samples were skimmed by centrifuging the samples at a speed of 4255 × *g* for 15 min at 10 ℃ using a Allegra X-30R centrifuge (Beckhman coulter, Inc, California, USA) and the separated fat layer was removed manually using spatula and muslin cloth. The process was repeated twice to achieve maximum removal of milk fats. Whey separation was achieved by isoelectric precipitation of casein proteins by reducing the pH as described by Kamal, et al.^9^. The samples were stored at 4 ℃ for 12 h to enhance casein precipitation. Casein precipitates were separated using centrifugation at 10,000 × *g* for 10 min at 4 ℃. Centrifugation was repeated three times to ensure efficient separation of casein from whey proteins. Protein content of the whey samples was determined using the Bicinchoninic acid method as previously described by Mudgil et al.^38^. Whey was stored at − 20 ℃ and freeze dried within 3 days of production using freeze dryer (Telstar, Spain). Lyophilized whey proteins were reconstituted into 150 ml of double distilled water adjusted to a final protein concentration of 3%.

### Camel whey protein hydrolysates generated with pepsin

The enzymatic hydrolysis of camel whey proteins (CWPs) was performed using gastric enzyme pepsin at pH 2.0 via a design of experiment (DOE) consisting of different process parameters (temperature, time and enzyme concentration). The DOE utilized CWP solution containing a protein concentration of 3% which was hydrolysed using pepsin at three temperatures of 30 ℃, 37 ℃, and 45 ℃; two hydrolysis time intervals of 120 and 360 min and three different enzyme concentrations (0.5, 1 and 2%) in a water bath shaker (Clifton NE5-28D, Nickel-electro LTD, North Somerset, UK) yielding a total of 18 different camel whey protein hydrolysates (CWPHs) (P1.1–1.3 to P6.1–6.3) as presented in Table 1. All hydrolysates produced at different conditions were generated in three batches. The enzymatic reaction was terminated by placing the samples in boiling water for 10 min followed by immediate cooling in ice cold water. Samples were then centrifuged at 10,000 × *g*, 15 min at 4 °C, supernatants were collected, pH was adjusted to neutral and stored at − 20 °C until later further analysis. The anti-cancer and anti-inflammatory activities were performed within two weeks of hydrolysis.

### Characterization of camel milk protein hydrolysate (CMPHs)

#### Degree of hydrolysis (% DH)

Degree of hydrolysis (DH) is defined as the proportion of cleaved peptide bonds in comparison to intact whey in a protein hydrolysate. It measures the free amino nitrogen (F-AN) using the O-phthalaldehyde (OPA) method of Church, et al.^39^. Briefly, fresh OPA reagent was prepared by mixing 25 ml of sodium tetraborate buffer (100 mM; pH 9.3), 2.5 ml of sodium dodecyl sulphate (20%, w/w), 40 mg of OPA (dissolved in 1 ml of methanol), and 100 µl of β-mercaptoethanol. The total volume was then raised to 50 ml using deionized water. A modified 96-well microplate assay was used, where 25 µl of samples were mixed with 300 µl of the OPA reagent and absorbances were measured at 340 nm, 30 min after incubation at 37 ℃. Simultaneously, a standard curve of tryptone was prepared and the free amino content was calculated. DH values of hydrolysates obtained were calculated using Eq. (1) as described by Kleekayai et al.^40^

1 $$DH\;\left( {\% } \right) = \frac{F - AN\;hydrolysate - F - AN\;intact\;whey\;protein \times 100 }{{Npb}}$$

Npb is the nitrogen content of the peptide bonds in whey protein (123.3 mg/g)^40^.

### Peptide profile by reverse phase-ultra performance liquid chromatography (RP-UPLC)

Reverse phase-ultra performance liquid chromatography (RP-UPLC) was utilized to monitor the peptide profile of the selected peptic camel whey protein hydrolysates^41^ with some modifications. Please refer to Supplementary material (S1) for details of the methodology.

### Anti-inflammatory activity assay

The anti-inflammatory activity, as indicated by the protection of thermal denaturation of albumin protein, was determined according to the method described by Aguilar-Toalá et al.^18^ and modified by Kamal et al.^9^. Please refer to Supplementary material (S3) for details of the methodology.

### Cell culture

The human liver cancer cell line, HepG2, was obtained frozen in liquid nitrogen from American Type Culture Collection (ATCC) and the human colon carcinoma cell line, HCT116, was obtained frozen from CLS Cell Lines Service GmbH. Both cells were cultured in defined RPMI 1640 medium (Gibco), supplemented with 10% fetal bovine serum (Sigma Aldrich) and containing 1% of 100 U/ml penicillin and 100 µg/ml streptomycin (Sigma Aldrich) at 37 °C in a humidified 5% CO_2_ atmosphere. Cells were sub-cultured each 3–5 days using trypsin 0.25% (Hyclone).

### Anti-proliferative assay

Effect of selected CWPHs (P-4.3 and P-5.2) on the proliferation of HCT116 cells was assessed using sulforhodamine-B (SRB) assay^42^. Cells were seeded in 96-well plates at a density of 8000 cells/well for 24 h and then treated with different concentrations of peptides. After 48 h incubation, cells were fixed with ice-cold 50% trichloroacetic acid, stained with 0.4% SRB and dissolved with 10 mM Tris base solution. Absorbance was taken at 564 nm using a microplate reader (Thermo scientific, USA). The IC50 was calculated using Graphpad prism.

### Cell cycle analysis

Cell cycle analysis was done using fluorescence-activated cell sorting (FACS) technique^42^. Cells were plated (5 × 10^6^) in 75 cm^2^ culture flasks. After 24 h, they were treated with CWPHs P-4.3 (231 μg/ml), incubated over a period of 24 h, 48 h and 72 h at 37 °C. After the incubation, cells were collected, fixed in 70% ethanol, treated with RNAse and stained with propidium iodide (1 mg/ml). DNA content was measured in FACScan flow cytometry (Becton Dickenson, San Jose, CA, USA) and data analysis were performed with FlowJo V.10 software (Tree Star, Inc, Ashland, OR, USA).

### Western blotting

Western blot analysis was performed as described previously^30^. Briefly, cells were treated with CWPHs (P-4.3, 231 μg/ml), incubated over a period of 24 h, 48 h and 72 h. The primary antibodies used were p53, p21, CDK1, p-CDK1 (Tyr 15), Cyclin B1, p-histoneH3 and GAPDH (Cell Signalling Technology). Further blots were treated with horseradish peroxidase–conjugated antibody. Protein bands were detected using WesternSure Chemiluminescent Substrate (LI-COR) and captured by a Versadoc imager system (Biorad, USA). Quantification of the proteins was performed using the ImageJ software with GAPDH as the internal control.

### Identification of peptides by liquid chromatography quadrupole time-of-flight tandem mass spectrometry (LC QTOF MS/MS)

CWPHs P-4.3 and P-5.2 were selected for peptide sequencing and identification ascribed to their potential anti-cancer activity against human liver cancer cell line (HepG2) (Supplementary Fig. S1). Peptide sequencing was conducted using liquid chromatography quadrupole time-of-flight tandem mass spectrometry (LC QTOF MS/MS, Agilent 6520) according to the protocol as described by Sarah et al.^43^. Please refer to supplementary material (S2) for details of the methodology.

### Physio-chemical properties of identified peptides and their in-silico structural activity relationship (SAR) within the active site of PLK-1 Polo box domain (PDB-ID: 3RQ7)

The list of peptides obtained were subsequently screened using Peptide Ranker web server available at http://distilldeep.ucd.ie/PeptideRanker/. Peptides with the score of above 0.50 were designated as potentially bioactive peptides (BAP) and subjected to a novelty check using a database search software (i.e. BIOPEP, PeptideDB, SwePep and EROP-Moscow). The MW, isoelectric point and net charge at pH 7 were calculated using a web utility called PepCalc (https://pepcalc.com/). Additionally, in silico prediction of the identified peptides, for their toxicity was done using the tool ToxinPred (https://webs.iiitd.edu.in/raghava/toxinpred/index.html), anti-inflammatory potential was checked using a sequence based prediction tool AIPpred (http://thegleelab.org/AIPpred/index.html) and anti-cancer potential was predicted using the ANTICP server (https://webs.iiitd.edu.in/raghava/anticp/index.html).

A web based program PepSite2 (http://pepsite2.russelllab.org/) was used to predict binding of a given peptide onto a protein structure (RCSB Protein Data Bank) to study the detailed interaction.

### Molecular docking

Molecular docking of selected peptides showing peptide ranker score of > 0.8 (AHLEQVLLR and ALPNIDPPTVER) was conducted against possible target protein obtained from the RSCB protein data bank (http://www.rcsb.org) using the MDockPeP server. Additionally, target prediction studies were carried-out using SwissTargetPrediction (http://www.swisstargetprediction.ch/), MolTarPred (http://moltarpred.marseille.inserm.fr/), SEA (http://sea.bkslab.org/) and PASS (http://www.pharmaexpert.ru/passonline/index.php) online search engines. The model with the highest ITScorePeP was considered for further analysis. The accuracy of the binding mode was assessed based on location of the original bound ligand. The 3D-best docked poses and interactions were visualized using PyMOL molecular viewer (Schrödinger Inc., New York, NY, USA).

### Statistical analysis

All the pepsin derived CWPHs were produced in three batches and degree of hydrolysis and anti-inflammatory activities were performed in triplicate and the data were subjected to Analysis of Variance (ANOVA) and appropriate means separation was conducted using Tukey’s multiple range test using a statistical software program (SPSS version 21 for Windows, 2012, Chicago, IL, USA). For anticancer activities, results are expressed as the mean ± SEM of at least three independent experiments and statistical analyses were performed using the Student's t-test.

## Supplementary Information

Supplementary Information.
