# Supplementary material for: Camel whey protein hydrolysates induced G2/M cellcycle arrest in human colorectal carcinoma
Source: Sci Rep. 2021 Mar 29;11:7062. doi: 10.1038/s41598-021-86391-z (PMC8007640; doi:10.1038/s41598-021-86391-z)

# **Camel whey protein hydrolysates induced G2/M cellcycle arrest in human colorectal carcinoma**

# Chandraprabha Murali^1^, Priti Mudgil^2^, Gan Chee Yuen^3^, Hamadeh Tarazi^4^, Raafat El-Awady^4^, Youssef Abdalla^5^, Amr Amin^1*^, Sajid Maqsood^2¥^

# ^1^Biology Department, College of Science, United Arab Emirates University, Al Ain, UAE

# ^2^ Food Science Department, College of Food and Agriculture, United Arab Emirates University, Al Ain, UAE

^3^Analytical Biochemistry Research Centre (ABrC), Universiti Sains Malaysia, 11800 USM, Penang, Malaysia.

# ^4^ College of Pharmacy, University of Sharjah, Sharjah, UAE

# ^5^Department of Kinesiology, Michigan State University, MI 48824, USA

**Corresponding authors:**

^*^ Amr Amin, Biology Department, UAE University, Al Ain P.O. Box 15551, UAE,

Tel.: +97137136519, [a.amin@uaeu.ac.ae](mailto:a.amin@uaeu.ac.ae)

^¥^ Sajid Maqsood, Department of Food Science, College of Food and Agriculture, UAE University, Al Ain P.O. Box 15551, UAE, Tel.: +97137134591, [sajid.m@uaeu.ac.ae](mailto:sajid.m@uaeu.ac.ae)

## ***S1. Peptide profile by Reverse phase-ultra performance liquid chromatography (RP-UPLC)***

Reverse phase-ultra performance liquid chromatography (RP-UPLC) was utilized for monitoring the peptide profile of the selected peptic camel whey protein hydrolysates [**^1^**](#_ENREF_1) with some modifications. For this, hydrolysates samples were briefly suspended in solvent A at a ratio of 1.5:10 *v/v* and vortexed vigorously for 2 min at room temperature. The samples were then filtered using 0.45 μm cellulose acetate syringe filters (Sigma-Aldrich, USA). The proteins and peptide profiles were eluted using a linear gradient of solvent A (0.1% TFA in HPLC grade water (*v/v*)) and solvent B (0.1% TFA in 80% acetonitrile; I in HPLC grade water). The flow rate was kept at 0.3 mL min^−1^, injection volume of 5 µL and separation of peptides and proteins were carried out at 25℃, using a 2.1 mm × 100 mm, 1.7 mm Acquity UPLC C18 BEH column (Waters, Milford, MA, USA). Solvent B was 0.05% (v/v) TFA in 60 % HPLC grade I in water. Whey proteins and peptides were eluted using a linear gradient of solvent B from 0 to 80% over 100 min. The absorbance of the eluent was monitored at 215 nm using photodiode array detector (Dionex UltiMate 3000 RS Diode Array detector).

## ***S2. Identification of peptides by liquid chromatography quadrupole time-of-flight tandem mass spectrometry (LC QTOF MS/MS)***

CWPHs P-4.3 and P-5.2 were selected for peptide sequencing and identification ascribed to their high anti-cancerous activity against liver cancer cell lines (HepG2 cells) (Supplementary Fig. S1). Peptide sequencing was conducted using liquid chromatography quadrupole time-of-flight tandem mass spectrometry (LC QTOF MS/MS, Agilent 6520) according to the protocol as described by [**Sarah, et al. ^2^**](#_ENREF_2). Briefly, 10 µL of injection volume was used and peptide separation was performed at a flow rate of 15 µL using AdvanceBio Peptide Map, C18 column (C18, 2.1 × 100 mm, 2.7 µm particles; Agilent, USA). The running gradient for HPLC separation system was performed using two mobile phase buffers namely solvent A (0.1 % formic acid in water) and solvent B (0.1 % formic acid in acetonitrile) as follows: 0-5 min, 10% (B); 5-115 min, 10-95% (B), 115-120 min, 95% (B), 120-135 min, 95%-10%, 140-150 min, 10% (B). The peptides were analysed using the electrospray ionisation-quadropole time-of-flight system (ESI-QTOF, Agilent 6520) with the following conditions: mass range: 100-2000 m/z; collision energy: 6V/100 Da (offset -2), flow rate: 15 µl/min; ion spray sources: 3.5 kV; drying gas: nitrogen at temperature of 350℃ with a flow rate of 10 L/min; nebuliser pressure: 3 psig; fragmentor voltage: 110 V, fragmentation mode: collision induced dissociation (CID). Peaks studio version 6.0 was used for data analysis with the average local confidence (ALC) set above 80% to ensure high data confidence.

## ***S3. Anti-inflammatory activity Assay***

The anti- inflammatory activity as indicated by the protection of thermal denaturation of albumin protein was determined according to the method described by [**Aguilar-Toalá, et al. ^3^**](#_ENREF_3) as modified by [**Kamal, et al. ^4^**](#_ENREF_4). Briefly, the reaction mixture (250 µL) consisted of 50 µL of fresh hen’s egg albumin, 100 µL of phosphate buffered saline (PBS, pH 6.4) and 100 µL of CWPHs samples in a 96 well microplate. The sample volume was replaced with equal volume of PBS that served as control. The reaction mixtures thus obtained were pre-incubated at 37℃ for 15 min and then heated at 70℃ for another 5 min. The absorbance was measured at 600 nm using a microplate reader (Thermo Fisher Scientific, Multiskan Sky, Vantaa, Finland). A standard curve of Diclofenac sodium (DS; reference ani-inflammatory drug) at the final concentration of 100 µg/mL-2500 µg/mL as positive control was prepared simultaneously. The results were calculated and expressed as DS Equivalent Capacity (DSEC)/mg of protein equivalent.

**Table S1. Peptides identified in hydrolysate P4.2 and P5.3 using liquid chromatography quadrupole time-of-flight tandem mass spectrometry (LC QTOF MS/MS)**

| **Peptide sequence for P-4.2** | **Peptide Ranker score** | **Peptide sequence for P-5.3** | **Pepsite Ranker Score** |
| --- | --- | --- | --- |
| **AHLEQVLLR** | **0.835698** | **LRPFL** | **0.93** |
| **ALPNIDPPTVER** | **0.823354** | **LRFPL** | **0.91** |
| AVLPFQEPVPDPVR | 0.758264 | LC | 0.84 |
| AVVSPIQF | 0.754309 | HSGF | 0.81 |
| AVVSPIQFR | 0.753343 | LWKL | 0.79 |
| DDVVIK | 0.74832 | PLLL | 0.74 |
| DFLK | 0.74315 | ESLPGVPPPSGQPLL | 0.74 |
| DILK | 0.740396 | KPVMECGALLL | 0.7 |
| DILKEDMPSQR | 0.691829 | CHLL | 0.68 |
| DLENLHLPLPLL | 0.68712 | CFNTLMLPPEPLL | 0.67 |
| DLENLHLPLPLLQ | 0.686639 | SHGM | 0.66 |
| DNENLQSR | 0.6636 | QVNLPLTGGML | 0.64 |
| DSALGLLR | 0.653826 | HSYAEEPPLYPLL | 0.63 |
| DV | 0.652205 | DLPLMT | 0.6 |
| DVQPTLSPGDR | 0.637388 | NNLPLTM | 0.58 |
| DVTVLDNTDGK | 0.626521 | PLMLPLHQPL | 0.57 |
| EDLVSK | 0.625677 | DLLPLTM | 0.57 |
| EDLVSKDDVVIK | 0.611102 | FLEL | 0.55 |
| EDLVSKDDVVIKS | 0.60455 | NHCDEPVPMT | 0.54 |
| ELAVVSPIQFR | 0.594786 | YTVF | 0.4 |
| ELTPGAATTLEGK | 0.593968 | HALL | 0.39 |
| ENIDELKDTR | 0.589707 | HSAM | 0.38 |
| ENLHLPLPLL | 0.588753 | SMLS | 0.36 |
| ENLHLPLPLLQ | 0.58596 | HLPTCLHESQPLL | 0.36 |
| EPIPYPILP | 0.581892 | YTFV | 0.34 |
| EPVPDPVR | 0.573527 | HDDTQPVPMT | 0.29 |
| EPVPDPVRG | 0.572758 | VVSEYFATVHGL | 0.26 |
| ETAAEVELR | 0.563312 | LLGV | 0.25 |
| ETIIPK | 0.562845 | TLLPK | 0.23 |
| ETMDFLK | 0.556007 | LHEM | 0.22 |
| FE | 0.549273 | LD | 0.22 |
| FLDDDLTDDK | 0.544486 | VVSNNGNSMSSPL | 0.2 |
| FLPPLQPAV | 0.535234 |  |  |
| FLPPLQPAVM | 0.529185 | HTESFPLMEVPEP | 0.18 |
| FPHASEVVKPQ | 0.528084 | HTESMLPLYPEP | 0.17 |
| FQEPVPDPVR | 0.523938 | YVPV | 0.16 |
| FRQENIDELK | 0.519923 | HVMS | 0.16 |
| FRQENIDELKDTR | 0.519127 | TVVSLDGMLCEGQA | 0.14 |
| GAHAGPTWNPISI | 0.514485 | TDTP | 0.11 |
| GLFQ | 0.51403 | TLTEGGAL | 0.09 |
| GLHPVPQP | 0.502458 | TVSTSLFSADT | 0.08 |
| GLHPVPQPLV | 0.50131 | EALSQ | 0.07 |
| GLHPVPQPLVP | 0.497465 | DEPE | 0.07 |
| GLHPVPQPLVPVI | 0.493812 | VVKPQ | 0.06 |
| GLHPVPQPLVPVIA | 0.493812 | EQMEEK | 0.05 |
| GLLRPFLDWTGPPEPLQK | 0.486723 |  |  |
| GYLAVAVVR | 0.486444 |  |  |
| HLEQVLLR | 0.482856 |  |  |
| HLPLP | 0.479692 |  |  |
| HLPLPLLQ | 0.467582 |  |  |
| HLPLPLLQS | 0.460765 |  |  |
| HMSGYDTETVVSNDGNR | 0.460765 |  |  |
| HMSGYDTETVVSNNGNR | 0.459534 |  |  |
| HPVPQESSF | 0.457113 |  |  |
| HPVPQESSFR | 0.455021 |  |  |
| HPVPQP | 0.451423 |  |  |
| HPVPQPLVPVIA | 0.446379 |  |  |
| HPYLEQLY | 0.439921 |  |  |
| HPYLEQLYR | 0.435313 |  |  |
| HTEPIPYP | 0.43226 |  |  |
| HTEPIPYPILP | 0.425266 |  |  |
| HTEPIPYPILPQN | 0.415783 |  |  |
| HTEPIPYPILPQNF | 0.413912 |  |  |
| IAHPSSYDTPEGIASEDGGK | 0.412731 |  |  |
| IAIPPK | 0.41271 |  |  |
| IAIPPK | 0.402515 |  |  |
| ILELAVVSPIQFR | 0.399982 |  |  |
| ILPQ | 0.393437 |  |  |
| ILTCLVAVAL | 0.384353 |  |  |
| INEDNHPQLGEPVK | 0.383586 |  |  |
| IVPR | 0.382022 |  |  |
| KEMPLL | 0.372589 |  |  |
| LAVAVVR | 0.357323 |  |  |
| LAVPINNQFIPYPNYAKPVAIR | 0.345494 |  |  |
| LAVPINNQFIPYPNYAKPVAIR | 0.342216 |  |  |
| LDDDLTDDK | 0.338827 |  |  |
| LENLHLPLPLL | 0.332362 |  |  |
| LENLHLPLPLLQ | 0.330565 |  |  |
| LHPVPQESSF | 0.328905 |  |  |
| LHPVPQESSFR | 0.327809 |  |  |
| LHPVPQPLVPVIA | 0.324066 |  |  |
| LLHPVPQE | 0.323142 |  |  |
| LLHPVPQESSF | 0.32087 |  |  |
| LLHPVPQESSFR | 0.313799 |  |  |
| LLQLEAIR | 0.311397 |  |  |
| LLRPFLDWTGPPEPLQK | 0.309681 |  |  |
| LPFQEPVPDPV | 0.302035 |  |  |
| LPFQEPVPDPVR | 0.297458 |  |  |
| LPFQEPVPDPVRG | 0.296988 |  |  |
| LPLPLL | 0.289294 |  |  |
| LPLPLLQ | 0.289294 |  |  |
| LPNIDPPTVER | 0.279868 |  |  |
| LPNIDPPTVER | 0.278086 |  |  |
| LPPLQ | 0.277346 |  |  |
| LPPLQPA | 0.276314 |  |  |
| LPPLQPAVM | 0.275694 |  |  |
| LPPLQPAVMV | 0.273986 |  |  |
| LPVPQQM | 0.270226 |  |  |
| LRPIAAEVY | 0.270226 |  |  |
| LSDELK | 0.263459 |  |  |
| LTDLENLHLPLPLL | 0.263459 |  |  |
| LTDLENLHLPLPLLQ | 0.262516 |  |  |
| LTLTDLENLHLPLPLL | 0.252303 |  |  |
| LTLTDLENLHLPLPLLQ | 0.250636 |  |  |
| LTPGAATTLEGK | 0.239626 |  |  |
| MDFLK | 0.238991 |  |  |
| MDVPK | 0.233577 |  |  |
| MVPYPQR | 0.231 |  |  |
| NEPTEDHIMEDTER | 0.220885 |  |  |
| NHQVSSEDLSMEPSISR | 0.21644 |  |  |
| NIDPPTVER | 0.2117 |  |  |
| NIDPPTVER | 0.210991 |  |  |
| NLENTMR | 0.203252 |  |  |
| NLHLPLPLL | 0.198657 |  |  |
| NLHLPLPLLQ | 0.197689 |  |  |
| NTATQSEETKELTPGAATTLEGK | 0.190481 |  |  |
| QALPNIDPPTVER | 0.179158 |  |  |
| QALPNIDPPTVER | 0.177691 |  |  |
| QENIDELKDTR | 0.172076 |  |  |
| QEPVPDPVR | 0.170905 |  |  |
| QLFGSPAGQK | 0.170905 |  |  |
| RPIAAEVY | 0.168927 |  |  |
| SALGLLR | 0.162983 |  |  |
| SHPYLEQLY | 0.159538 |  |  |
| SHPYLEQLYR | 0.158406 |  |  |
| SHTEPIPYP | 0.157085 |  |  |
| SHTEPIPYPILP | 0.156196 |  |  |
| SHTEPIPYPILPQN | 0.152205 |  |  |
| SHTEPIPYPILPQNF | 0.147581 |  |  |
| SLFPHASE | 0.146637 |  |  |
| SLFPHASEVVKPQ | 0.143268 |  |  |
| SLTLTDLENLHLPLPLLQ | 0.1381 |  |  |
| SPVVPFT | 0.136511 |  |  |
| STVFESLPAK | 0.135074 |  |  |
| TAAEVELR | 0.132264 |  |  |
| TDLENLHLPLPLL | 0.1321 |  |  |
| TDLENLHLPLPLLQ | 0.13013 |  |  |
| TEPIPYPILPQN | 0.124278 |  |  |
| TIIP | 0.12384 |  |  |
| TIIPK | 0.12384 |  |  |
| TKETIIPK | 0.122578 |  |  |
| TLTDLENLHLPLPLL | 0.118816 |  |  |
| TLTDLENLHLPLPLLQ | 0.117655 |  |  |
| TMDFLK | 0.113234 |  |  |
| VAHLEQVLLR | 0.112471 |  |  |
| VLPFQEPVPDPV | 0.104182 |  |  |
| VLPFQEPVPDPVR | 0.096916 |  |  |
| VLPFQEPVPDPVRG | 0.0955286 |  |  |
| VLPVPQQ | 0.094283 |  |  |
| VNPFQEPVPDPVR | 0.0921602 |  |  |
| VPFLQPK | 0.0906955 |  |  |
| VPQESSFR | 0.0834116 |  |  |
| VPQPLVPVIA | 0.0824839 |  |  |
| VPVIA | 0.0816745 |  |  |
| VPVLA | 0.0810969 |  |  |
| VPYPQR | 0.0780098 |  |  |
| VPYPQR | 0.0750801 |  |  |
| VVSPIQFR | 0.0632251 |  |  |
| VYSHTEPIPYPIL | 0.0529294 |  |  |
| YLEELHR | 0.0461662 |  |  |
| YPEVFQNEPDSIEEVLN | 0.0461662 |  |  |
| YPLR | 0.0461662 |  |  |

**Figure S1**

***
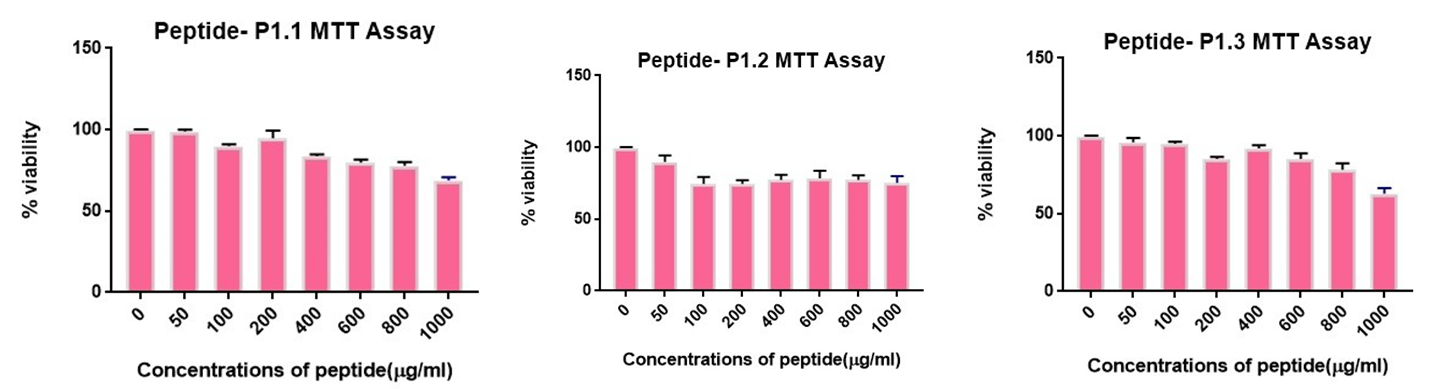
***

***
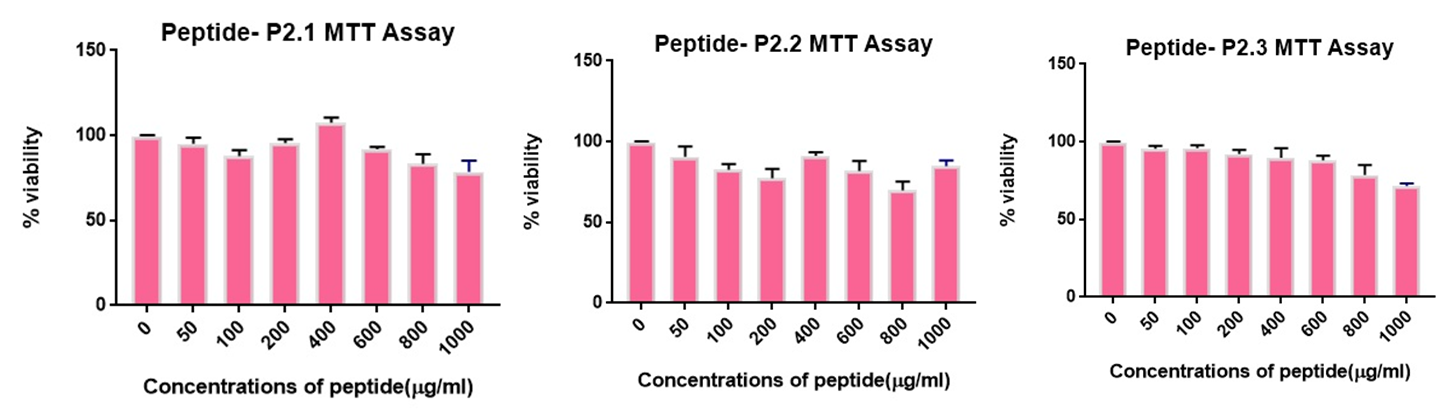
***

***
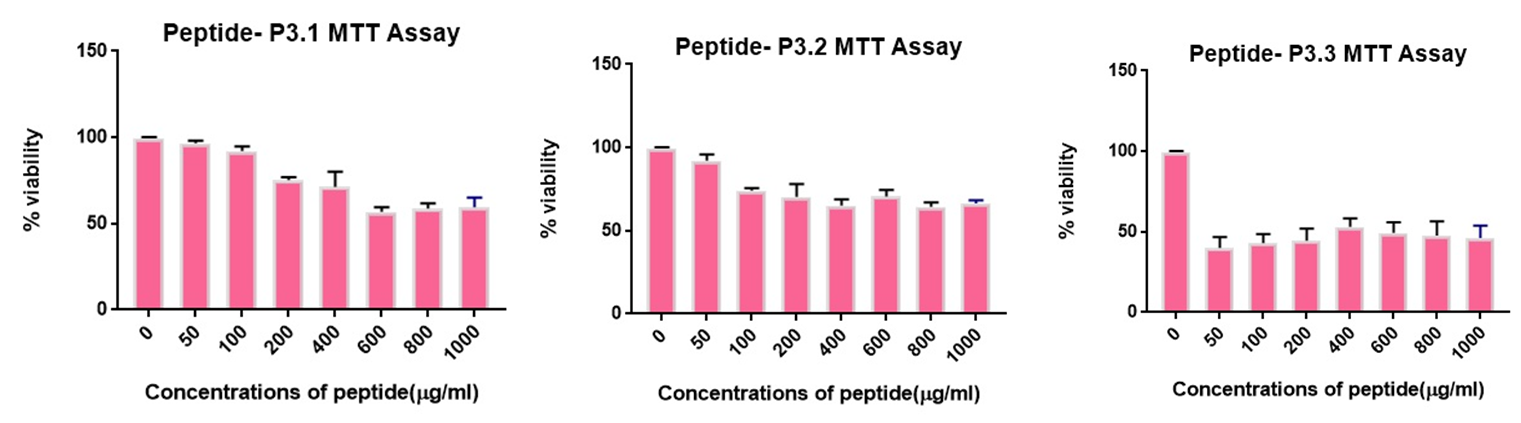
***

***
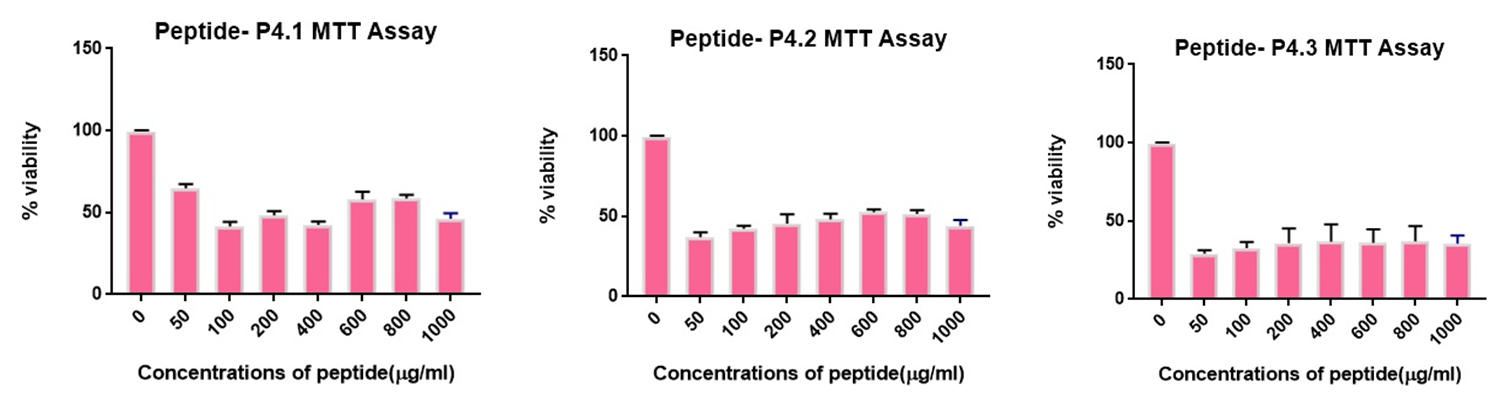
***

***
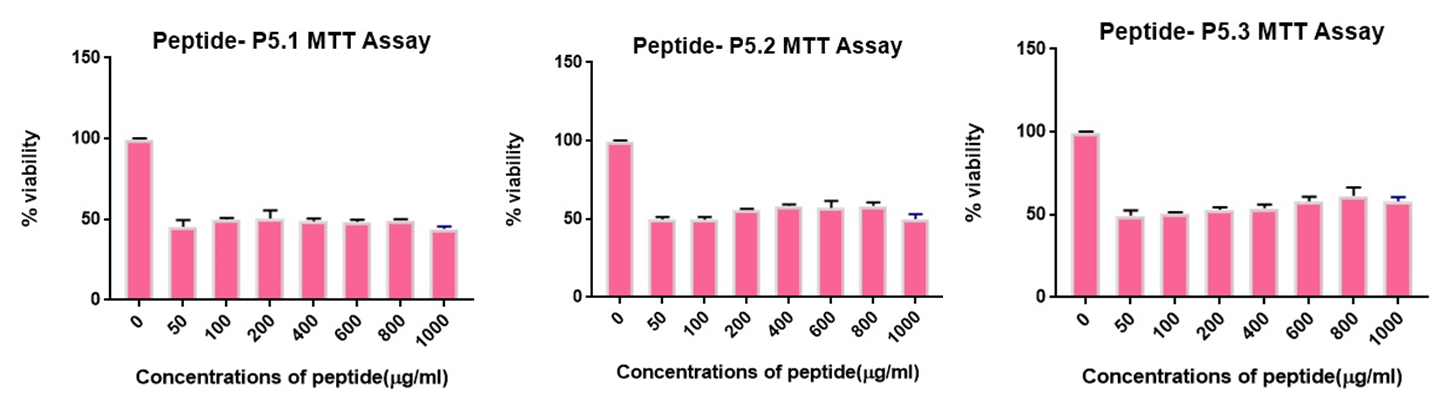
***

***
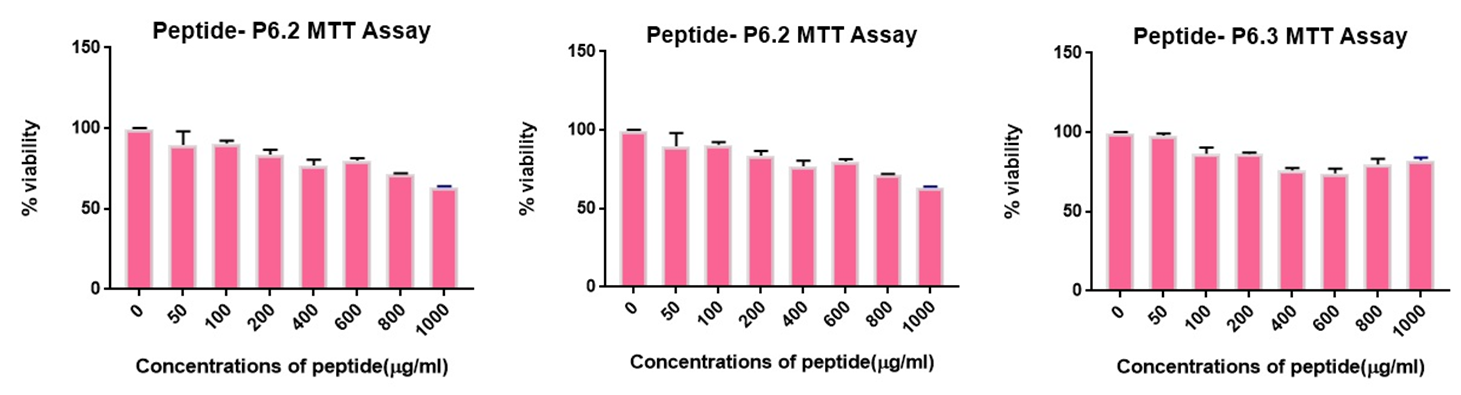
***

***Figure S1. Camel peptides screening on the Growth and Survival of HePG2 Cells.*** Cell viability of HePG2 cells after treatment with increasing concentrations (50-1000ug/ml) of the various camel peptides for a period of 24 h**.** Among the peptides, **P3.3, P4.1, P4.2, P4.3, P5.1, P5.2, P5.3** were the peptides that showed 50% or more growth inhibitory effect.

## **References**

1 Nongonierma, A. B. & Fitzgerald, R. J. Tryptophan-containing milk protein-derived dipeptides inhibit xanthine oxidase. *Peptides* **37**, 263-272, doi:10.1016/j.peptides.2012.07.030 (2012).

2 Sarah, S. *et al.* LC–QTOF-MS identification of porcine-specific peptide in heat treated pork identifies candidate markers for meat species determination. *Food Chemistry* **199**, 157-164 (2016).

3 Aguilar-Toalá, J. *et al.* Assessment of multifunctional activity of bioactive peptides derived from fermented milk by specific *Lactobacillus plantarum* strains. *Journal of Dairy Science* **100**, 65-75 (2017).

4 Kamal, H. *et al.* Inhibitory properties of camel whey protein hydrolysates toward liver cancer cells, dipeptidyl peptidase-IV, and inflammation. *Journal of Dairy Science* **101**, 1-10 (2018).


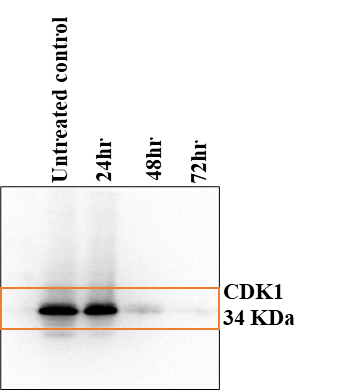

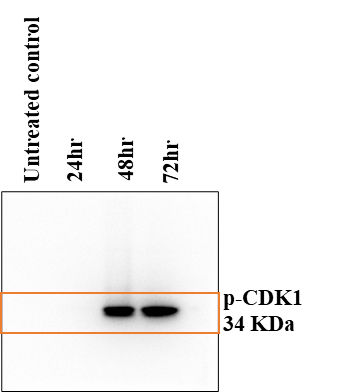

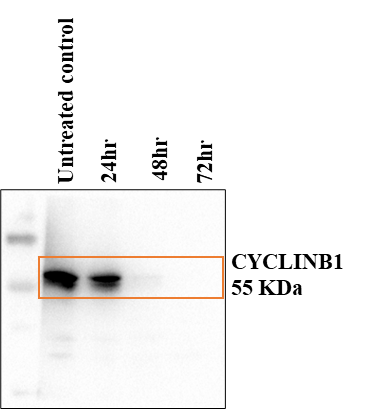

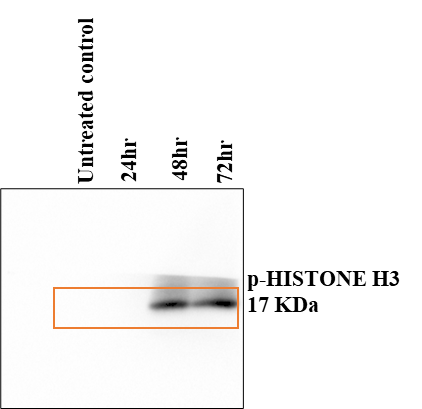


**(a)**

**FIGURE 2**


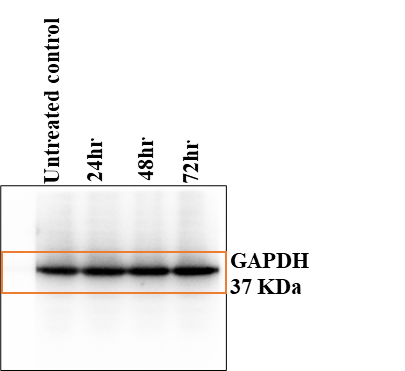


**FIGURE 2**

**(c)**


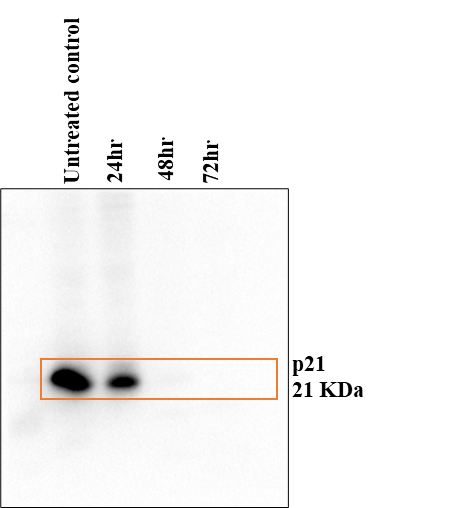

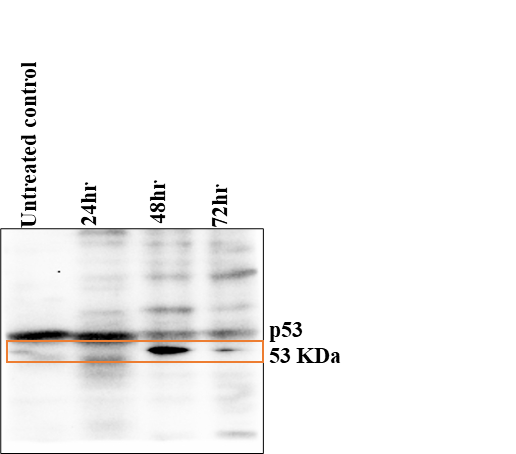

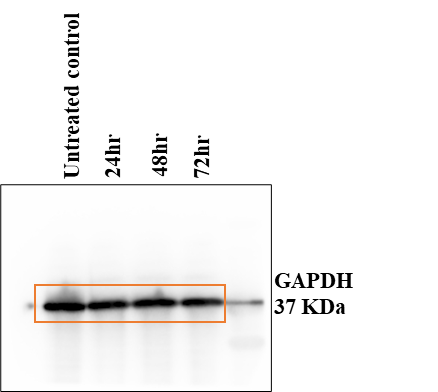

Supplement: Supplementary file 1 — Supplementary Information. [file 41598_2021_86391_MOESM1_ESM.docx]
